# Sustaining Translational Research

**DOI:** 10.5041/RMMJ.10409

**Published:** 2020-10-14

**Authors:** Sir Robert Lechler

**Affiliations:** King’s College London, London, UK

## INTRODUCTION†

Good morning. It is a great pleasure to share my thoughts with you here in Israel.

The last day I was here was June 25, 2016, which was the day of the results of the Brexit referendum. My wife and I escaped feeling so depressed, but at least, we thought, we are leaving this behind; but when we came to Israel the only thing people wanted to talk about was the Brexit referendum!

Here I am today, on October 31, 2019, the day that we were meant to be leaving the European Union, so there is something with my connection with Israel and Brexit that I don’t understand!

Let me start by saying that we have the privilege of being participants in a biomedical and health science revolution (Slide 2, Additional Material‡). I don’t think that is overstating it. We are living in an extraordinary, exciting time. And let me just remind you of some of these fields that are moving so fast.

You know about the omics explosion and the sequencing of the first human genome. That took 10 years, it cost $3 billion dollars, and now we can do a genome in a day for $600 dollars, and that continues to be a falling price. And that is just genomics, and there is all the other omics coming along.

And then is the field of gene editing, which is controversial because of some activities in China. But there is undoubtedly going to be an impact on a number of disease states, particularly those with single-gene disorders, with somatic cell gene editing and replacement.

That of course is paving the way for precision medicine, and that is well developed in cancer, but now extending to other disease areas such as diabetes.

The digital revolution was rather slow to penetrate healthcare but now is undoubtedly having a big impact, and I’ll come back to that.

The brain is beginning to reveal some of its secrets. Indeed, if I was starting again, I think I would probably do neuropsychiatry because I think it’s going to be such an exciting area of medicine.

And finally, my own territory of immunotherapy. A number of cancers that 10 years ago we thought were untreatable are now treatable. And, indeed, the other side of the coin: in autoimmune diseases and transplantation, we are making real progress in turning the immune system off—as well as in cancer, turning it on.

It’s an incredibly exciting time, and we are very fortunate to be working in this field right now.

The other point I would make by way of introduction is that many of us, and I’m sure many of you in this room, have grown up working in mouse models. They’ve been very useful and informative, but, increasingly now, we can think of the human being as the experimental animal of the twenty-first century because we can do so many more things in patients than used to be the case.

But just in case you thought the job was done, it’s not done! There are some huge challenges that we yet have to address. There hasn’t been a new class of psychiatric drug for 30 years; we have no effective treatment for dementia; no new class of antibiotics for 30 years; no success in promising tissue regeneration *in situ*; and the pandemic of obesity marches on. And there are many other challenges that no doubt you are grappling with yourselves.

What I want to go through quite quickly is what I regard to be the key steps we collectively need to take if we are going to accelerate the translation of discovery into patient benefit. I’m not going to spend much time on these, though I will spend a bit of time on the first one, which is maintaining a balanced science base.

## MAINTAINING A BALANCED SCIENCE BASE

Now, in the UK, and I’m sure it’s true in many other parts of the world, we keep an eye on the balance of research funding that goes to discovery science versus applied science. Slide 7 shows a slightly out-of-date look at how much funding went to underpinning science, basically discovery science, versus to translational research. You will see that there has been a shift towards translation. Some would say that is a good thing; some would say, “Oh my gosh, basic science is at risk.”

If you ask the question, “is curiosity-driven research an unaffordable luxury?”, I think Carl Sagan, who was an American political writer, made an interesting remark. That “cutting off fundamental, curiosity-driven science is like eating the seed corn. We may have a little more to eat next winter but what will we plant so we and our children will have enough to get through the winters to come?”

So, the way that I think about this is informed by … oh before I come to that …

I’m sure you all have your own favorite examples of where curiosity-driven research has led to very important subsequent applications. So, the people investigating nuclear magnetic resonance had no idea of its potential for clinical scans at all, and they won the Nobel prize for it, and of course it has opened up a new field of medicine.

When transistors were discovered they were viewed as “lab curiosities” with no practical use.

Taq polymerase and green fluorescent protein (GFP) were purely driven by curiosity-driven research and turned out to be very important in cell biology.

And then the first person to clone a gene from *E. coli* did it simply to see if it could be done, and then warned against the dangers of genetic engineering! And so, we’ve moved on from there.

Anyhow, I think a helpful way to frame this question in this debate was provided by Dr Donald Stokes, who was head of a school of political science in the USA, and he described a very simple 2×2 quadrant where the vertical axis is how *fundamental* some research is, and the horizontal axis how *practical and applied* it is (Slide 10). He gave examples in each case. For pure *basic* research, he chose Niels Bohr, the Danish particle physicist (Slide 11). He had no interest in application, but of course some of what he discovered turned out to be applicable. For *applied* research, his poster child was Thomas Edison, the serial inventor who died with 1,096 patents to his name (Slide 12). And for what he called “use-inspired” basic science, his poster child was Louis Pasteur (Slide 13). The one quadrant you don’t want to be in is the one in the bottom left, which is boring and useless science, but I’m sure there is no one in this room who fits into that quadrant!

My argument would be that, for leading research institutions or research environments such as the one that you have here, the thing we need to do is to make sure that all three of these quadrants are occupied, that they are all thriving, but they are connected. I think if you do that, then we have nothing to worry about. But it is important that all three are sustained.

Now if you think about whether Donald Stokes was scoring marks for diversity: choosing three white, old men, I don’t think he was, but we’ll forgive him that.

The language I use is creating a research environment where scientists have “line of sight” all the way from discovery through to application. If I took you to King’s College London where I am in charge of all the health faculties, the basic scientists there would say “I like being here because, even though I am not a translational researcher, I know that down the corridor there are people interested in translation research who will take what I discover and apply it, if it is applicable”. The key is a close relationship between the researchers and the users of the research, the healthcare system, patients, commercial entities, and the drivers of research. I think if we do that, we will get the balance roughly right.

And, of course, all of this drives what we call in the UK the academic health science center model, and I am going to come back to that in a moment, because that is about linking the scientific push with the clinical pull so that you get the best of both.

Now, you may or may not have heard of the Francis Crick Institute, opened a couple of years ago behind St Pancras station in London (Slide 16). It is a wonderful building, and it has some wonderful science going on there inside, led by Paul Nurse, Nobel Prize winner, and they just got another Nobel Prize awarded to Peter Ratcliffe for his work on oxygen-sensing genes. But if you ask the question, “is this just another discovery science institute?”, I would say, emphatically not. And the reason is that the unique thing about the Crick is that the three major London universities and their academic health science centers (AHSC)—King’s, University College London (UCL), and Imperial—are partners in the Crick (Slide 17). We each invested 40 million British Pounds of capital into the building. That gives us each 80 body spaces to occupy in the building, and this provides three conduits for translational research from this powerhouse of discovery into these clinical environments. To my knowledge this is a unique experiment and one I am very optimistic will work well, provided that some of these basic scientists take the risk of coming out to work on our academic medical campuses, to explore translational opportunities as and when they arise.

So that is the first point.

## FOSTERING UNIVERSITY–HEALTHCARE PARTNERSHIPS

The second point that is absolutely essential is that we foster really close partnerships between universities and healthcare organizations.

The background to this in the UK is that these two entities, two sectors, have drifted apart over the last few decades, for a variety of reasons (Slide 19). One is that the healthcare system has become very preoccupied with targets (nothing wrong with targets by the way); they’ve had a good effect in some ways, and led by management rather than clinicians (again, nothing wrong with good managers, they are very important people), but that means that the R&D agenda has been secondary rather than primary. Turning to the university sector, we have something in the UK called the research excellence framework (https://www.ref.ac.uk/), and it is easier to generate high-quality research in basic science and in animal models than it is in the clinical context, and so perhaps there has been a greater focus away from clinical research, and the financial pressures, again, tend to drive organizations to focus on their immediate priorities rather than on what is important in the long term.

Now, I don’t know whether this little joke is going to cross national boundaries, and so forgive me if it doesn’t. And I guess people here know of Winnie the Pooh and they’ve read stories in their childhood. Well, Pooh has a very, very good friend, and here they are (Slide 20) on a woozle hunt wandering around looking for a woozle. While the two friends wandered through the snow on their way home, Piglet grinned to himself thinking how lucky he was to have a best friend like Pooh. Now this cartoon came around the Internet at the time the swine flu virus was identified. And Pooh was thinking less charitable thoughts to himself, and forgive the language, it’s not mine, “If the pig sneezes, he’s fffing dead” (Slide 21). And so, the swine flu virus put at risk this very long-standing relationship. And I think our relationship between universities and healthcare in the UK, at least, has been under threat for those reasons that I have just mentioned.

For these reasons, the UK decided to launch a competition to designate a small number of academic health science centers (Slide 22). There are now six of these across the UK, and they are designed to bring universities and their hospital partners into a much more intimate relationship in order to drive high-impact innovation into improved clinical outcomes in the population.

Let me now tell you a little about the academic health science center that I lead (Slide 23). It is called King’s Health Partners, and it brings three hospitals together, Guy’s and St Thomas’ (GSTT), King’s College Hospital (KCH), and the UK’s leading mental health trust, South London and Maudsley (SLaM), with a university partner, King’s College London (KCL). In my view, to be a twenty-first century academic health science center, you need to tick three boxes as a baseline: Firstly, you need to provide excellence in clinical care, excellence in research, and excellence in education. Secondly, you need to be broad in your range of services and research, and thirdly you need scale.

I think we can claim all three of those. We certainly are large. We have 36,000 staff and have a turnover getting close to £4 billion per annum, with 5 million patient contacts and a very large research portfolio.

There are three hypotheses underlying what we are trying to do in King’s Health Partners, derived from the partners in this organization (Slide 24). So, if I’m talking to some innocent bystander at a bus stop, this is how I would explain what King’s Health Partners is about.

The first hypothesis is that by having two large acute hospitals, GSTT and KCH, in the same partnership, there must be an opportunity to *reconfigure specialist services* and link the relevant research and increase quality. It’s a lot easier to say than it is to do, as I am sure you well know. The second hypothesis is that by having a mental health hospital in this partnership, we should be able to do something to better *integrate mental and physical healthcare*. And the third hypothesis is that by having a university in this partnership, we should be able to *create an academic culture and accelerate translation*.

Let me briefly illustrate whether or not we are making progress with delivering on those three hypotheses.

### Reconfiguration of Specialist Services

My presentation (Slides 25–27) provides a diagram showing the specialist services delivered by GSTT. There are two hospitals, one organization on two sites, and KCH is three miles down the road. You don’t need to look at this very long to see that all these specialist services are duplicated. Now, if you were designing the healthcare system from scratch you would not design it like this, and that is why I call this a dog’s breakfast—it’s a mess. When we established King’s Health Partners, we saw an opportunity to do something more intelligent about it. Now, as I say, it’s an awful lot easier to say “Let’s reconfigure things” than it is to do it because nobody wants to give up what they have.

But over the last 10 years we have managed to make a lot of progress, and now we have committed consensually to developing a series of clinical academic institutes consolidating cardiovascular and child health at St Thomas’, cancer and dentistry at Guy’s, and neuroscience, diabetes, and hematology at Kings College Hospital. So, we are reconfiguring services. We’ve done this in a very fair and balanced way, and I think it has a lot of potential through driving improved clinical quality and translational research.

So that’s hypothesis one.

### Integration of Physical with Mental Healthcare

Hypothesis two—I describe this as putting an end to Cartesian dualism. To understand that, you need to understand a little bit of philosophy. There was a French philosopher called Descartes who believed in the duality of the mind and the body—thought they were two separate things. Philosophy has largely moved on from that thinking, but our healthcare systems have not. And so, at Denmark Hill, KCH is on one side of the road, the SLaM hospital on the other side of the road, and the relationship between these two wasn’t very good in the past, and I’m told you needed a passport to cross the road when going from one hospital to the other!

This was not sensible. And this is something I’ve become passionate about since leading King’s Health Partners. The three drivers of this passion are shown on Slide 28, and I’m sure you know this.

First, by our own screening at King’s Health Partners, almost one-third of patients with long-term physical conditions are depressed. While rheumatologists are very good at looking after joints, they are not so good at detecting or managing depression. The same thing for diabetes and so on.

Secondly, 60% of patients referred to a cardiologist with chest pain have nothing wrong with their heart. That doesn’t mean they don’t have chest pain, but it means they get investigated inside and out until someone realizes that the problem is anxiety.

And thirdly, patients with long-term serious mental illness—and this is the worst scandal of all—have about 17 years taken off their lifespan. This is not suicide; this is the physical comorbidities that accompany their schizophrenia, and that’s partly due to drugs, partly due to lifestyle.

We need to do something about this, and this is a major theme across the whole of King’s Health Partners (Slide 29). We are looking to do everything we can to integrate across these boundaries, and so we now have 58 outpatient clinics which are co-staffed by a physician and a psychologist. We screen patients waiting in outpatients for mental health issues, we are training our mental health nurses to recognize insulin resistance and hypertension, and so on. I think this is immensely important.

### Translation of Research into Clinical Practices

What about hypothesis three: are we managing to integrate the university into the healthcare system and generate an academic culture? Slide 30 shows the number of highly cited papers published by NHS employees in our hospitals. These are not university employees; they are NHS employees. King’s Health Partners was formed between 2008 and 2009. Slide 31 shows that publications have trebled in GSTT and doubled in KCH. I can’t prove that’s due to the Academic Health Science Center, but I am going to claim it as a credit.

Then if you look at clinical trial performance, which we measure across all hospitals in the UK, GSTT is now either first or second, KCH fourth and eleventh, and SLaM first or third (Slide 31). This is the clinical trial performance across the whole of the UK, and ten years ago, that was absolutely not the case. These are surrogate markers, but I hope you will accept that they do provide some encouragement—that we are generating a really academic research-orientated culture.

## ESTABLISHING A SUSTAINABLE HEALTHCARE SYSTEM

The third challenge is establishing a sustainable healthcare system, because if our healthcare systems are not financially secure, there is very little chance that they will be able to prioritize research. In the UK that is a real challenge at the moment. I think all of that drives this concept of value-based healthcare, which I’m sure is alive and well here (Slide 33). But what we mean by value-based healthcare is measuring outcomes that matter to patients, service users, and their carers, divided by the resources, including the costs, of achieving those outcomes over the complete pathway of care. That is the value equation, and we need really to focus on that if we are going to have any chance of a sustainable healthcare system.

This is another major crosscutting theme across the whole of our academic health sciences center (Slides 34–35). All of our clinical academic groups—that is the major clinical research constellations—have to produce outcomes books, moving on to much more dynamic outcomes scorecards; we do lots of communications around this theme. We work with local partners and so on.

And I have a whole other talk on this, which I’m not going to give you this morning. But these are four categories of value-creating interventions that I can evidence for you in proving clinical outcomes at lower cost. And they are what I’ve talked about: Service reconfiguration, pathway redesign, frugal innovation, and the most important value-based proposition of all, which is prevention. If we can manage to capitalize on some of the discoveries that I’ve referred to earlier, for example, from genomics, polygenic risk scores identifying the high-risk sectors of the healthy population where we need to target interventions, I think those are the things we need to do if we are going to have a sustainable healthcare system.

## FOSTERING PARTNERSHIPS WITH INDUSTRY

Fourthly, fostering partnerships with industry. And that is something that I know you do exceptionally well in this country, and I think it is really important that we continue to drive that. Because the old model of drug development is broken (Slide 37). That’s when pharmaceutical companies used to have their own R&D on their own premises, and it became less and less successful; the returns on R&D investment in the pharmaceutical industry just steadily declined, and the cost of getting a drug to market has steadily increased (Slide 38). And so, we need new models, and that is happening in the UK, and I know it’s also happening here.

Pfizer has its experiment with centers for translational innovation where they are embedding Pfizer research groups in hospital environments; AstraZeneca is doing the same sort of thing, and GSK likewise (Slide 39).

Slide 40 shows the Addenbrookes site in Cambridge where AstraZeneca is putting its whole R&D headquarters right alongside the Addenbrookes Hospital. I think these are models that we need to continue to drive, and of course we are trying to do that at King’s in London.

In order to foster that more intimate relationship between industry and academia and healthcare, I think there is a series of steps that we can take (Slide 41). I think we need to involve industry in our undergraduate (UG) teaching; I think we need internships so it’s much easier to move between sectors; sabbaticals I think are a very good mechanism too. I think a much more porous boundary between our sectors is what we need to be successful.

This is all about partnerships (Slide 42). We need partnerships between academic institutions, that is higher education institution (HEI) and HEI; we are not very good at that. We tend to compete very often at the expense of collaboration. In London we are working very hard at getting better at collaborating. We then need partnerships between academia and healthcare, and then partnerships again with industry. If we get this right, then we will get a tri-partite relationship to deliver a tri-partite mission.

In the UK, to finish this section, we are moving towards life sciences clusters springing up around the country (Slide 43). Again, it’s something that you have done very well here in Israel. I think this is an intelligent way to organize how things are shaped in a geographical region.

## ATTRACTING THE MOST ABLE CLINICAL AND NON-CLINICAL SCIENTISTS INTO BIOMEDICAL RESEARCH CAREERS

This fifth point is absolutely crucial (Slide 44): and that is making sure that we attract the most able scientists, both clinical and non-clinical, into biomedical research careers. I don’t know how it is in this country, but it is vulnerable in the UK. The career pathway is insecure, and we are at risk of losing people, not least, actually, because of the Brexit effect in terms of European scientists.

I think we also need to pay attention to diversity in our workforce. Slide 45 shows the gender data from the UK. It just shows you how wrong we are getting it because if you look at clinical academics or non-clinical academics, at the junior lecturer level you can see it’s roughly 50–50 male–female, but in the professoriate it drops to about 20% women, and there is clearly something wrong there, unless you are daft enough to think that women are less intelligent—then we are simply wasting resource by not making it easy for women to progress through academic ranks. And then we get into ethnic diversity where the data are even worse.

The only other comment I would make is something that struck me when I was here three years ago, that it’s that my understanding that universities do not tend to fund clinical academic salaries for research-orientated physicians. You’ve got fantastic research institutions like the Weizman and so on, you have fantastic hospitals, like Rambam here, but universities are actually not choosing to fund the salaries of clinician-scientists, which I think is something that you might well think about addressing.

## BUILDING AN EXPERIMENTAL MEDICINE INFRASTRUCTURE

The sixth point is (Slide 47): it’s vital to build the infrastructure for what I call “experimental medicine,” because that biomedical revolution I talked about earlier is entirely dependent, if it’s going to be translated, on having really first-class safe facilities for doing early-phase trials with these novel therapies in patients.

We have been working very hard on that on our campus. Slide 48 illustrates the Guy’s campus (GSTT). I don’t know whether you are aware of “The Shard,” the tallest building in Europe I believe, that has risen at London Bridge, dwarfing the Guy’s Tower, which was quite tall before The Shard was put up beside it. The Shard has also cosmetically challenged the Guy’s Tower, which wasn’t the prettiest building, so it’s had a facelift, but this building is turning itself, floor-by-floor, into an experimental medicine facility. And I’ve just seen a diagram of your Discovery Tower that you are building here, and I’m sure that it will be something similar. I’m not going to waste time going through each floor. But we have every kind of facility that you would want in order to carry out, safely, first-in-man studies of advanced therapeutics, for example, including GMP [good manufacturing practices] facilities, a genomics core, etc.

On the St Thomas’ campus (Slide 49), which is the one opposite the houses of Parliament, there we are developing a very exciting med-tech hub where we are bringing biomedical engineering right into the heart of the hospital, and the plan is to create a London Institute of Healthcare Engineering; we have got 450 imaging scientists there, and we are broadening out to other aspects of biomedical engineering over the next decade, led by a very talented young Frenchman, Seb Ourselin. The thing that I am particularly excited about here is that the engineering is really embedded in the hospital context, and I think that is key. We need to bring disciplines into medicine outside biomedical research if we are going to make the progress that we wish to make.

## ANTICIPATING THE EVOLUTION OF HEALTHCARE: THE DIGITAL REVOLUTION

A penultimate point is to really get engaged with the digital revolution, which I’m sure you are, and we are endeavoring to do so (Slide 50). It is going to have a very big impact; it already is (Slide 51): the possibility now of this technology for remote monitoring, which is actually having some very early dividends in low- and middle-income countries where you can monitor retinal disease or skin lesions, really in very primitive settings, by transferring the data to some central monitoring source. But it is also relevant to the way we organize our healthcare systems, and hopefully this will lead to less dependence on trips to hospitals because we will be able to manage patients in their home setting much more efficiently. Lots of connectivity of data sets (Slide 52). I’m sure you are grappling with the data deluge here in Israel. But it really has huge potential to extend our insights into disease pathogenesis and personalized medicine. The applications of artificial intelligence are already having an impact in assisted diagnoses and disease monitoring (Slide 53).

Bearing that in mind, we need to think a little bit more creatively about how we are generating a workforce that can embrace some aspects of this revolution that I started off talking about (Slide 54). We need to be breeding many more data scientists, informaticians, and machine-learning experts that are going to be comfortable working in the clinical context. I think it also has implications for how we train doctors, and not only doctors, but other healthcare professional groups too.

## ENGAGING THE PUBLIC EFFECTIVELY

And my final point is public engagement (Slide 55). It is absolutely vital that we take this aspect of our responsibility seriously. I am sure, as in the UK, when you complete your grant application, there is a little section at the end where you have to say what you are going to do about informing the public or disseminating your discovery, and we do it as a kind of duty, but we don’t take it very seriously. I think we need to take it more seriously.

The reason for that (Slide 57) is that we need the public’s permission (is the language I use) to do the research that we do, because ultimately the public are the people that fund us through their taxes, one way or another. As the biomedical revolution progresses, it is throwing up a number of issues with ethical dimensions, and we need the public to engage with those dimensions, so, as I say, that we keep them on side as agents working with us as we continue to prosecute the research that we want to do.

## CONCLUSION

To bring all this to a finish (Slide 58), what I’ve suggested to you is that if we are going to harness this revolution that I’ve started off talking about, we need to maintain a balanced science base so as to continue supporting discovery science; we need to foster university–healthcare partnerships so as to create line of sight from discovery to translation; we must bring academic rigor forward to establish a sustainable healthcare system; more porous boundaries between academia and industry must be created to attract clinical and non-clinical scientists into biomedical research—including addressing the gender gap; we must invest in the building of key medical infrastructure; anticipation of the future and being prepared for the digital revolution is critical; and finally engaging the public effectively must be prioritized.

I have not talked about this, but I think it is also important that we go on demonstrating to our government the economic value of research, which I’m absolutely convinced is the case. Thank you for staying with me and thank you for your attention.

## Supplementary Information



## Footnotes

† This is a transcript of the Rambam Grand Rounds Lecture given on October 31, 2019 at Rambam Health Care Campus, Haifa, Israel. The actual lecture with all slides can be viewed on the Rambam Maimonides Medical Journal Channel of YouTube at: https://youtu.be/MG64qWZ4UIw

‡ All slide numbers refer to the slides in the additional material.

## Abbreviation

GFP: green fluorescent protein
GSTT: Guy’s and St Thomas’
HEI: higher education institute
SLaM: South London and Maudsley
KCH: King’s College Hospital
KCL: King’s College London
UCL: University College London

