# Supplementary material for: Sustaining Translational Research
Source: Rambam Maimonides Med J. 2020 Oct 14;11(4):e0035. doi: 10.5041/RMMJ.10409 (PMC7571437; doi:10.5041/RMMJ.10409)
Supplement: Supplementary file 1 [file rmmj-11-4-e0035am.pdf]

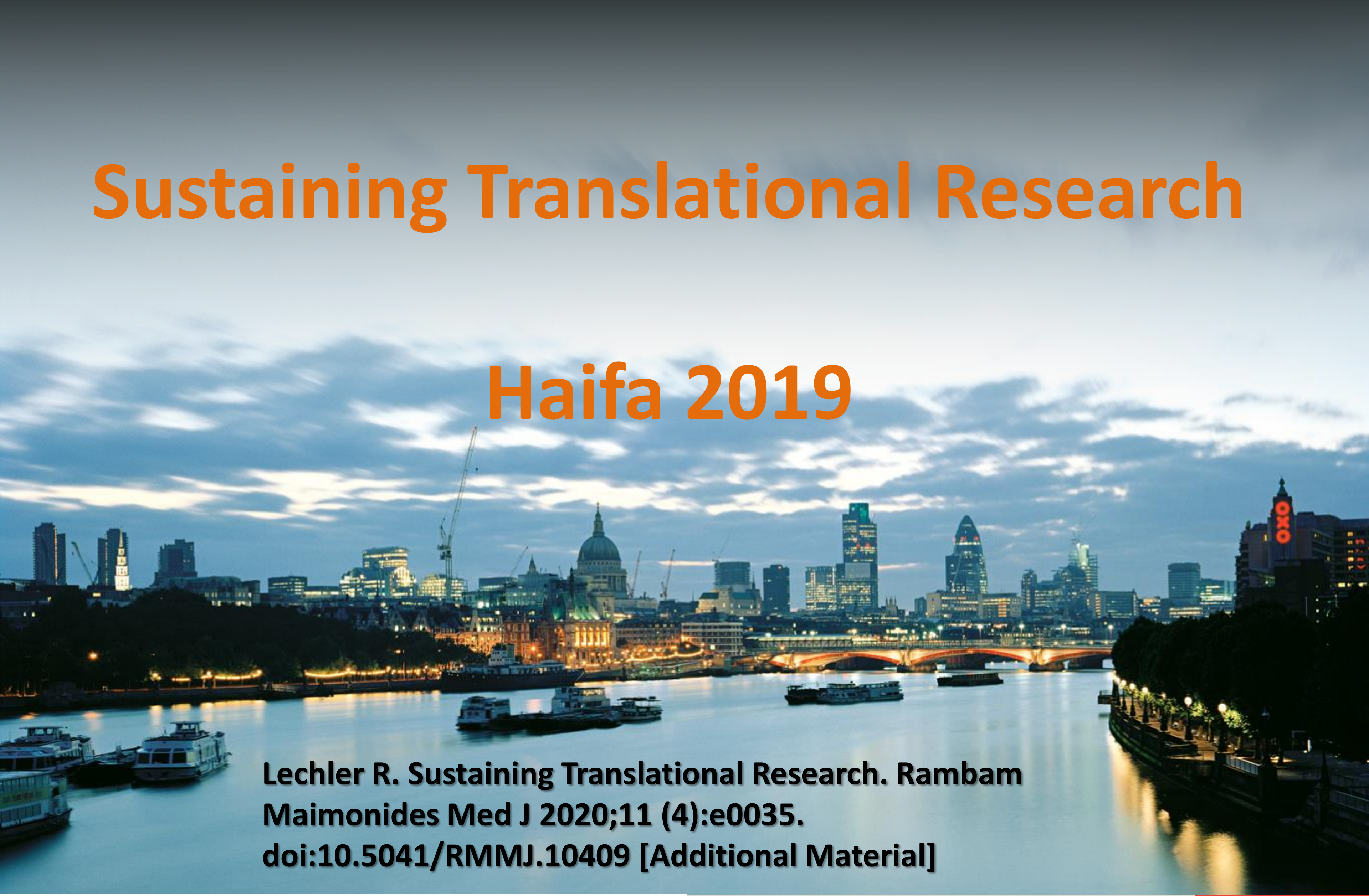

Sustaining Translational Research

Haifa 2019

Lechler R. Sustaining Translational Research. Rambam Maimonides Med J 2020;11 (4):e0035. doi:10.5041/RMMJ.10409 [Additional Material]

KING'S HEALTH PARTNERS
Pioneering better health for all

Robert Lechler

KING'S College LONDON

A biomedical and health science revolution

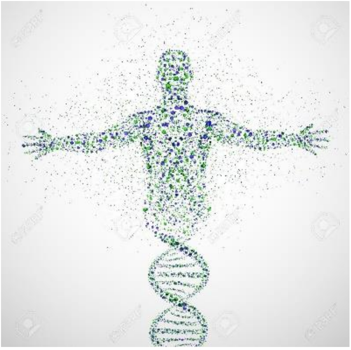

'Omics explosion

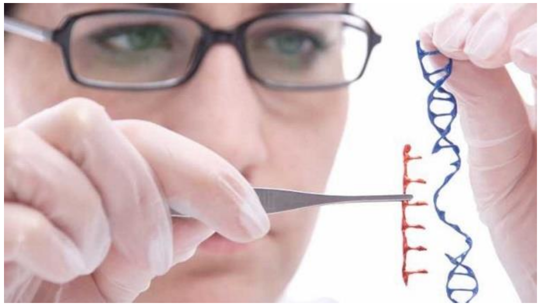

Gene editing & replacement

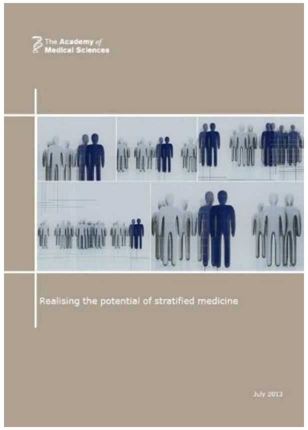

Stratified/precision medicine

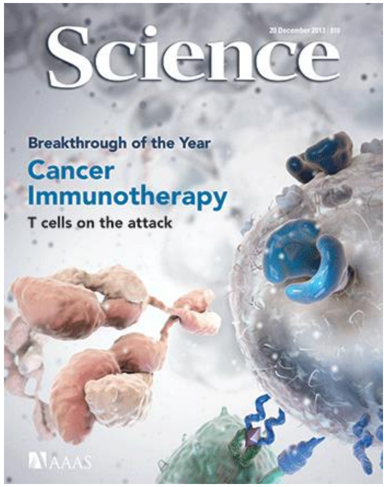

Cancer immunotherapy

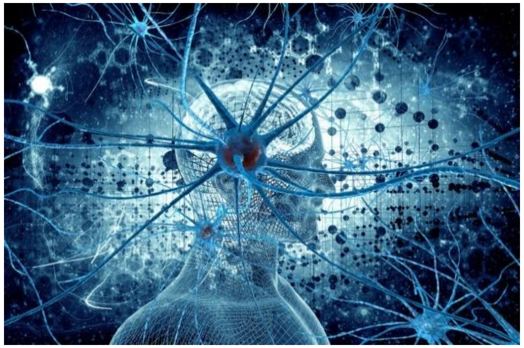

Insights into biology of psychiatric disease

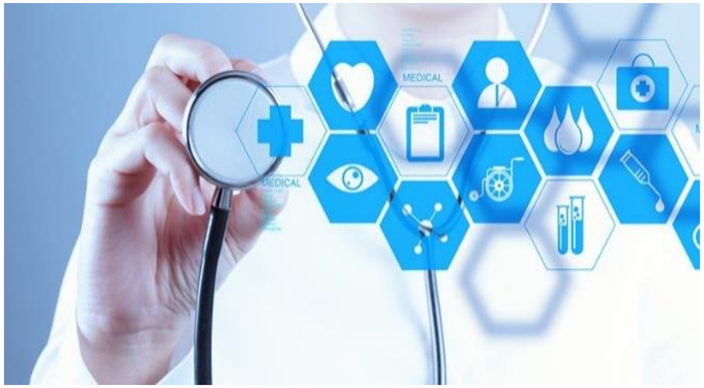

Digital technology

Experimental medicine

“In the last 50 years we have been given all the tools we need in order to bring the investigations to ourselves” Sydney Brenner

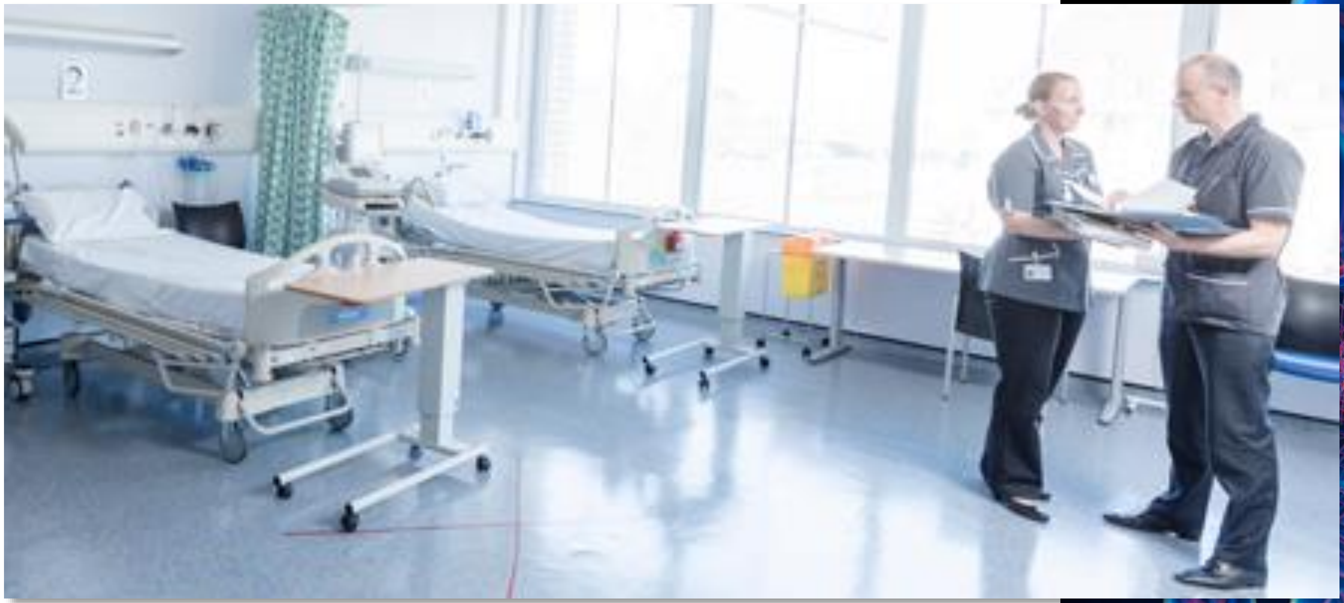

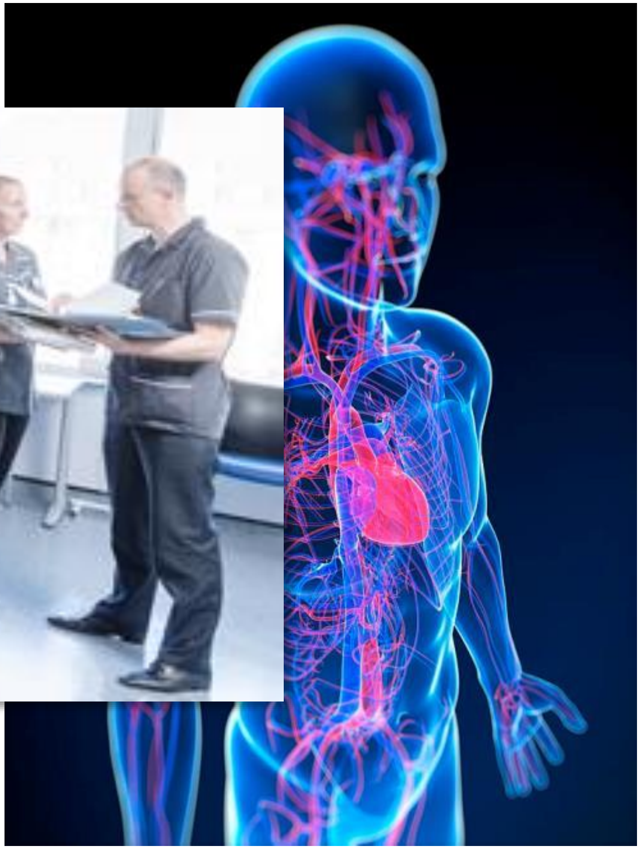

Major challenges remain:

- No new psychiatric drugs for 30 years
- No effective treatment for dementia
- No new class of antibiotics for 30 years (except Teixobactin)
- No success in promoting tissue regeneration *in situ*
- The pandemic of obesity marches on

Keys to accelerating translation

- Maintaining a balanced science base
- Fostering university – healthcare partnerships
- Establishing a sustainable healthcare system
- Fostering partnerships with industry
- Attracting the most able scientists, clinical and non-clinical, into biomedical research careers
- Building the infrastructure for “experimental medicine”
- Anticipating the evolution of healthcare – the digital revolution
- Effective public engagement

Keys to accelerating translation

- Maintaining a balanced science base
- Fostering university – healthcare partnerships
- Establishing a sustainable healthcare system
- Fostering partnerships with industry
- Attracting the most able scientists, clinical and non-clinical, into biomedical research careers
- Building the infrastructure for “experimental medicine”
- Anticipating the evolution of healthcare – the digital revolution
- Effective public engagement

Changes in Research Activity 2004-2014

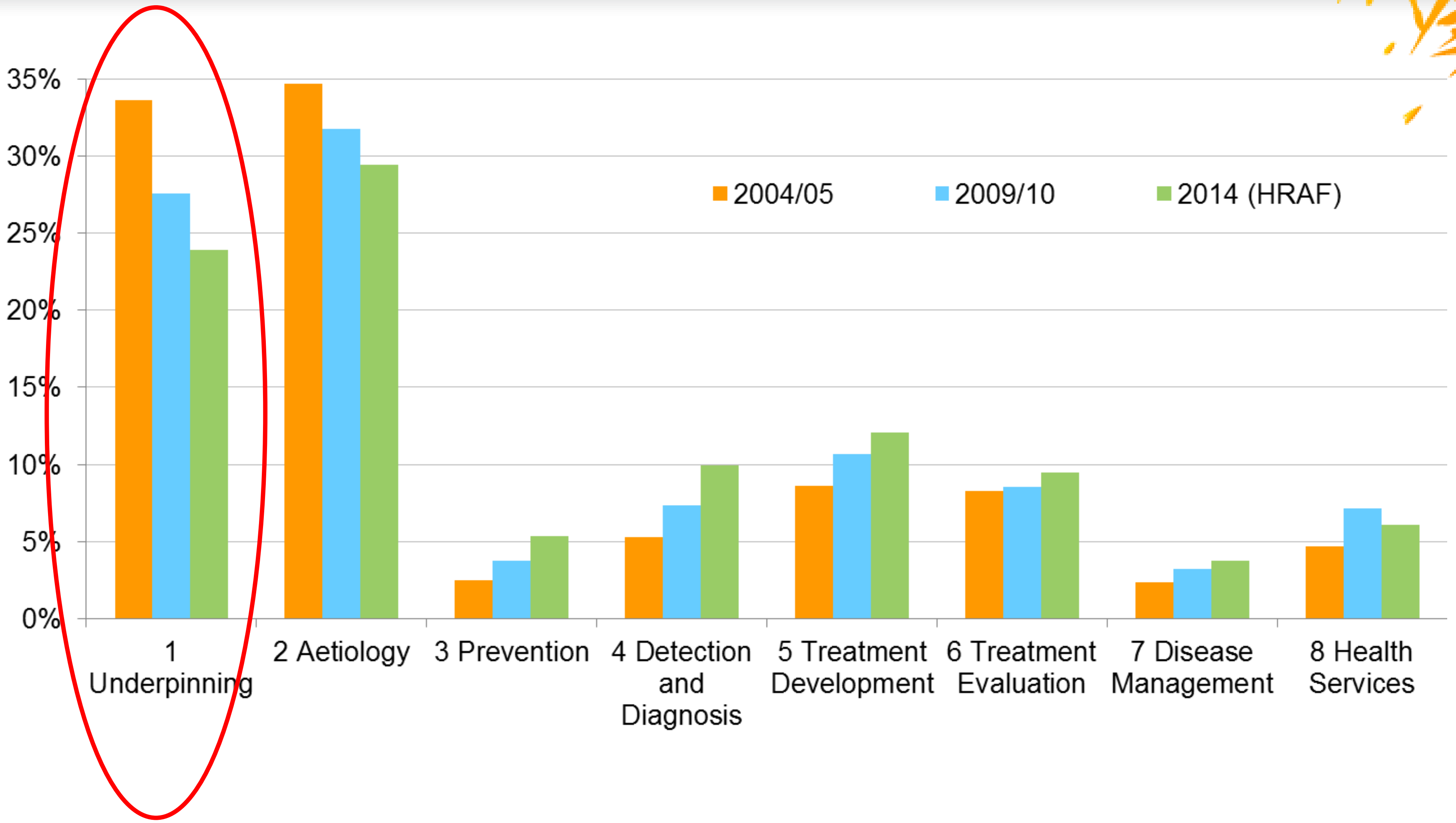

Working in partnership. Changing cultures. Igniting our potential

Biomedical science: is curiosity-driven research an unaffordable luxury?

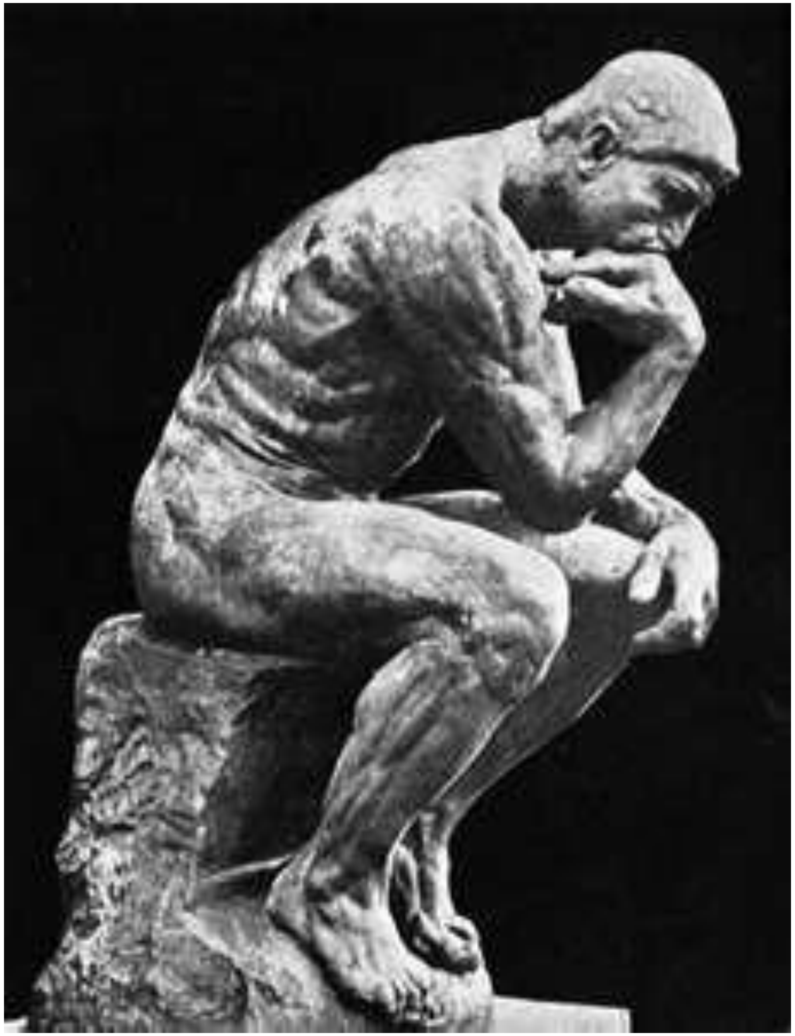

"Cutting off fundamental, curiosity-driven science is like eating the seed corn. We may have a little more to eat next winter but what will we plant so we and our children will have enough to get through the winters to come?" Carl Sagan

Curiosity-driven research leads to unexpected impact

- Nuclear magnetic resonance - clear that Nobel Prize Winners Bloch & Purcell had no idea of the possibility of MRI

- Transistors – viewed as “lab curiosities” with no practical use

- Taq polymerase and GFP – essential experimental tools

- John Beckwith isolated the first gene from E. coli in 1970 simply to see if it could be done. Following this achievement he warned against the dangers of genetic engineering!

Stoke's (Pasteur's) Quadrant

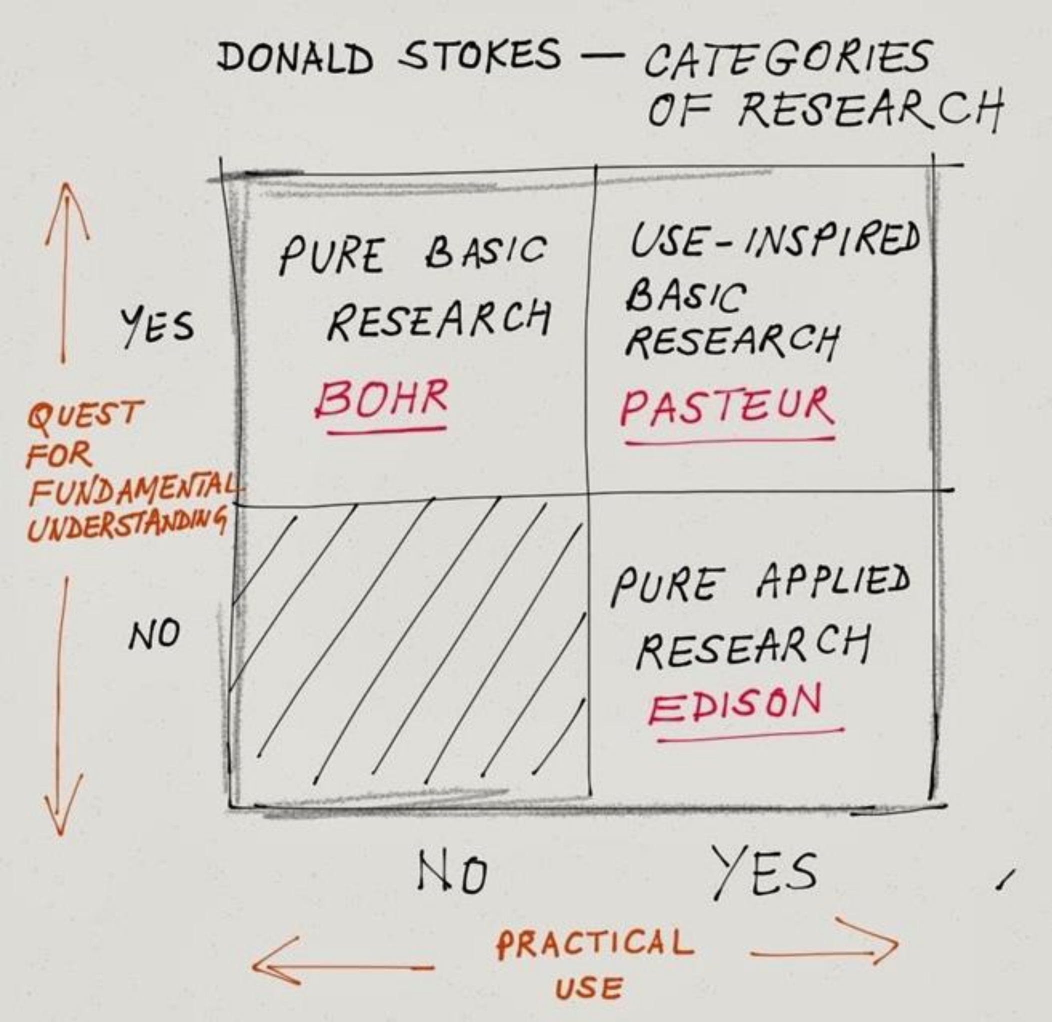

Stoke's (Pasteur's) Quadrant

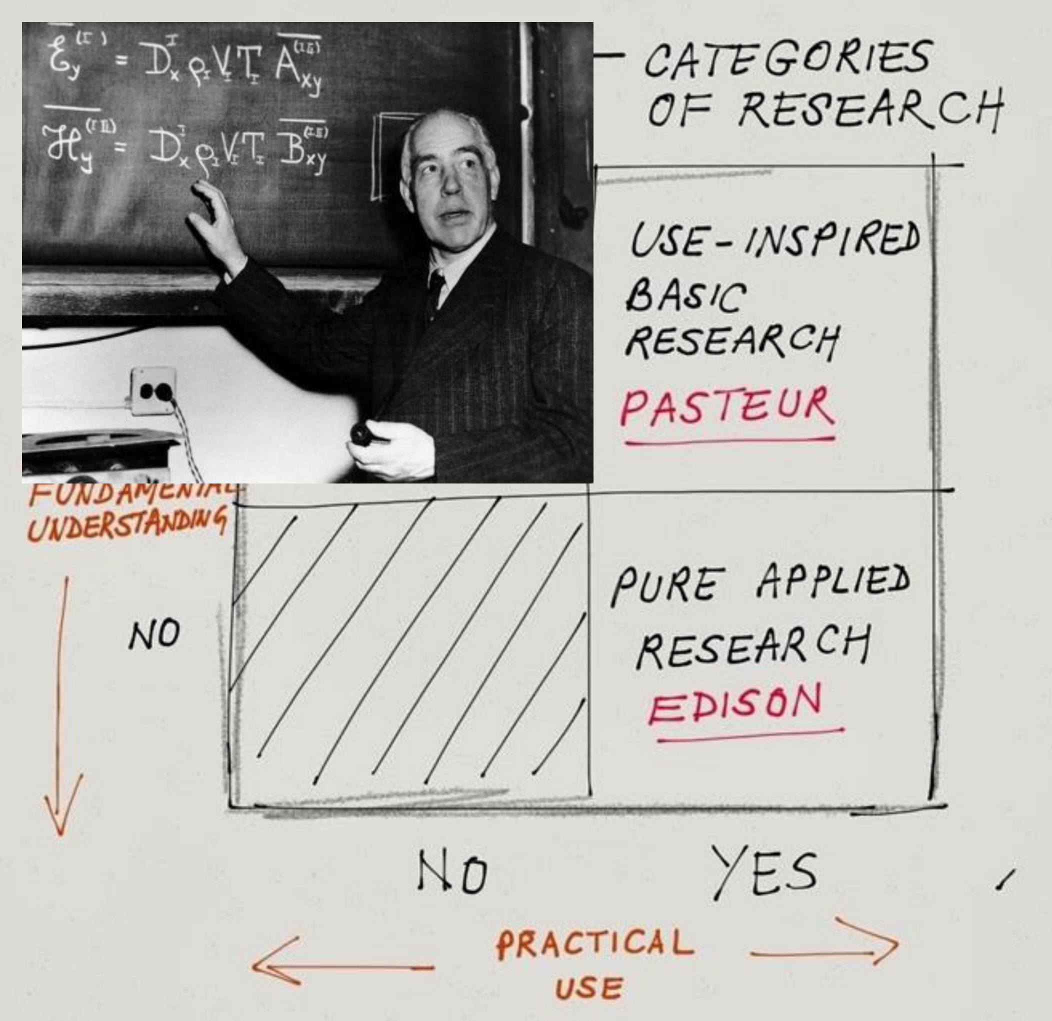

Stoke’s (Pasteur’s) Quadrant

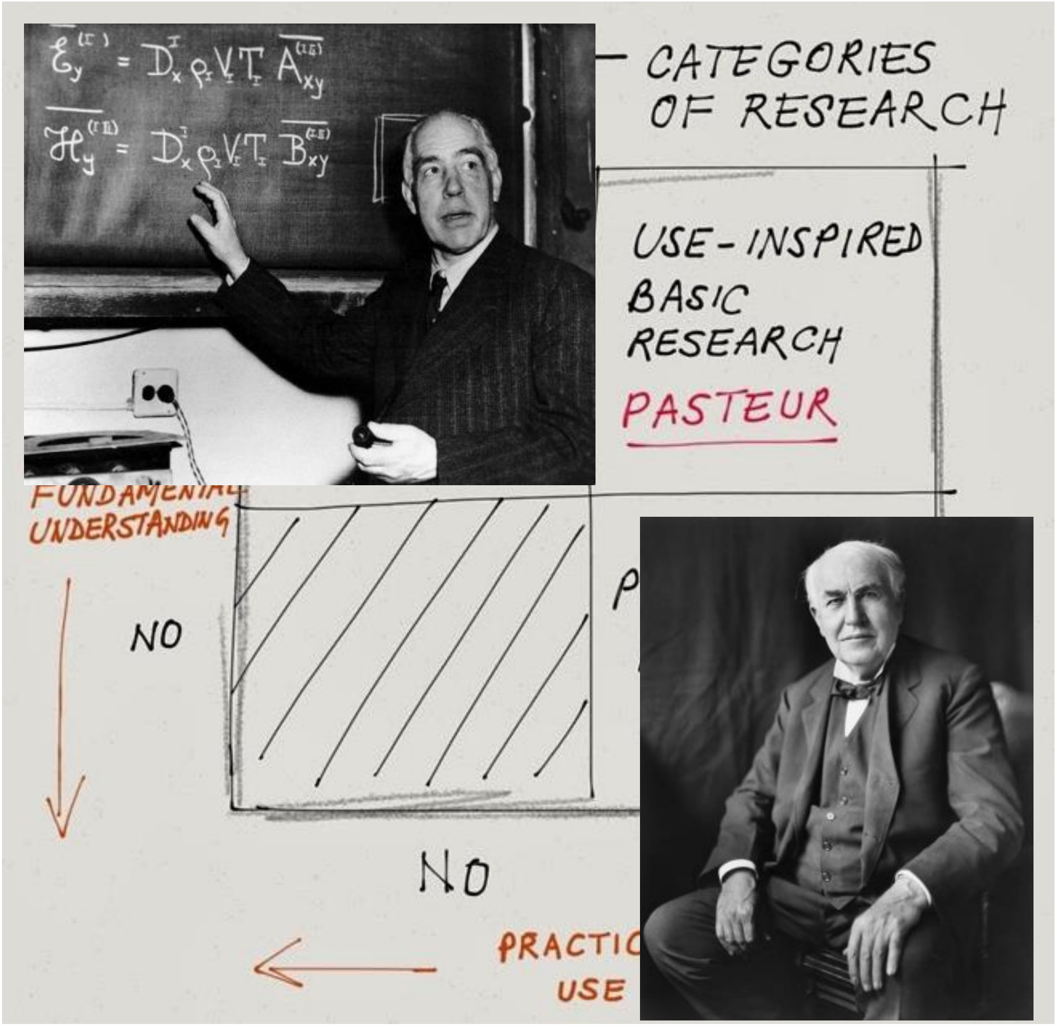

Stoke’s (Pasteur’s) Quadrant

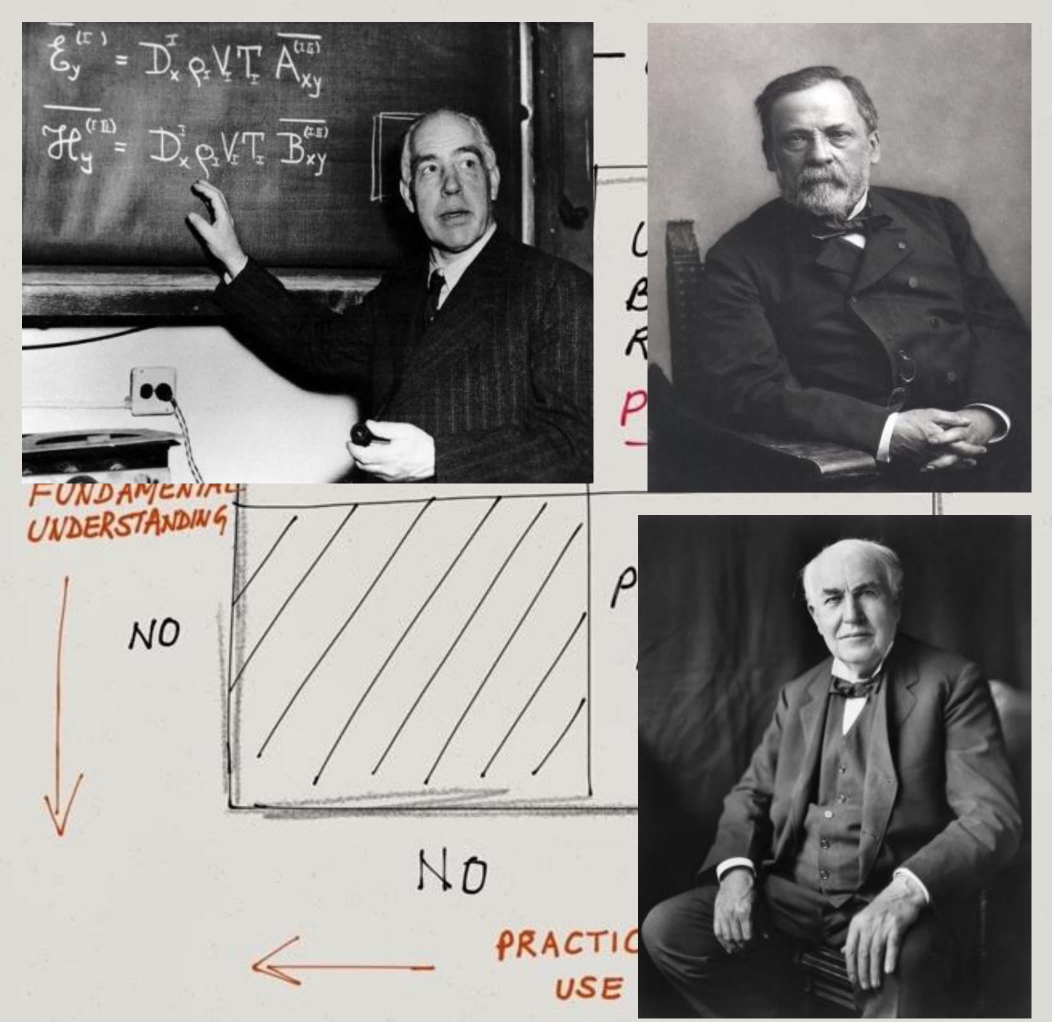

diversity…!

Impact in a clinical academic environment

- Culture: "line of sight" from discovery to application

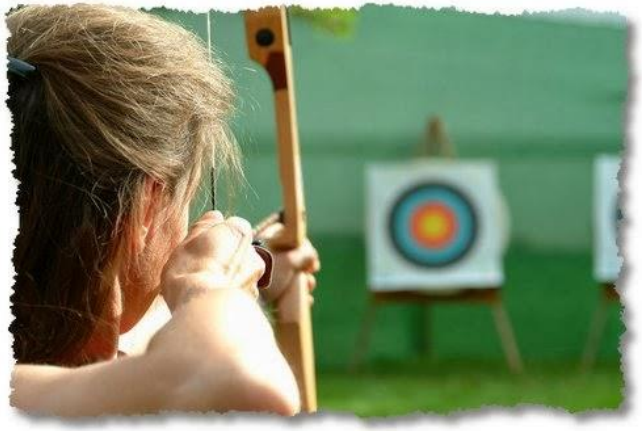

- Close relationships with "users" of research: NHS, patients, business, cultural industries, MoD…
- Specific partnerships with pharma, biotech, medtech
- Policy impact important (e.g. alcohol)

The Academic Health Science Centre model

- Continuum of research from discovery science, through experimental medicine, to clinical trials and health services research
- Scientific “push” linked to clinical “pull”
- Shared infrastructure: Biomedical Research Centres, Clinical Research Networks
- *Current evidence suggests that there is an association between the engagement of individuals and healthcare organisations in research and improvements in healthcare performance* (Boaz A *BMJ Open* 2015)

Francis Crick Institute

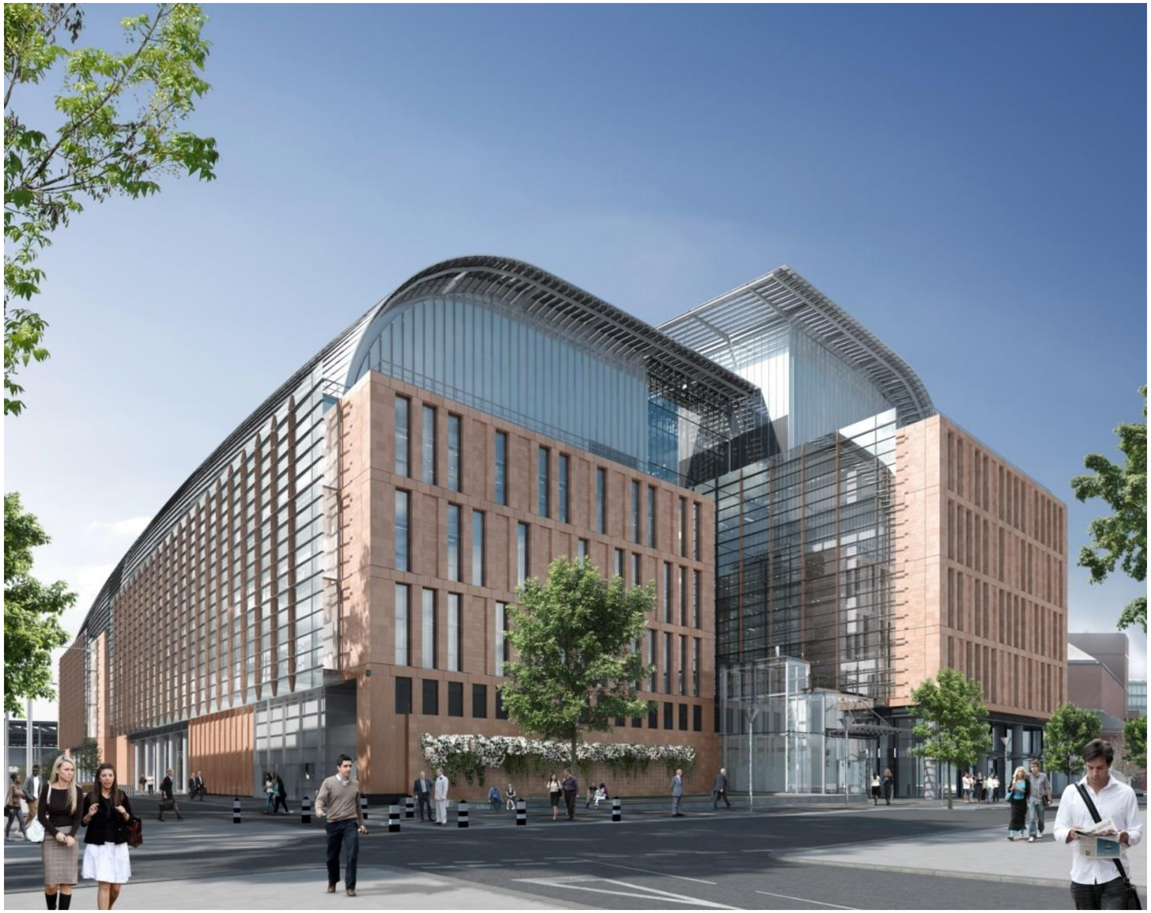

Another discovery science institute…?

Powerhouse of discovery science linked to three conduits for translation

Imperial

University and AHSC

UCL

University and AHSC

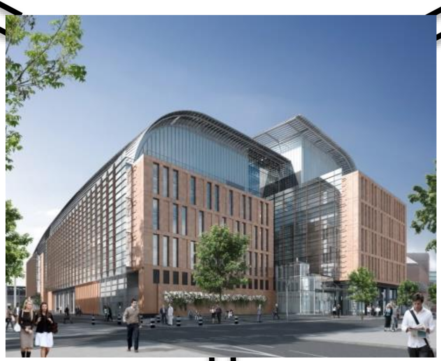

King’s

University and AHSC

Keys to accelerating translation

- Maintaining a balanced science base
- Fostering university – healthcare partnerships
- Establishing a sustainable healthcare system
- Fostering partnerships with industry
- Attracting the most able scientists, clinical and non-clinical, into biomedical research careers
- Building the infrastructure for “experimental medicine”
- Anticipating the evolution of healthcare – the digital revolution
- Effective public engagement

Universities and their hospital partner in the UK have drifted apart in recent years

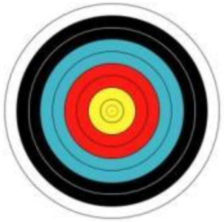

the target and management culture of the NHS

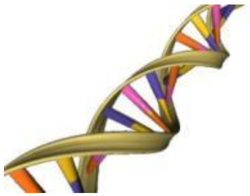

The REF's emphasis on basic science

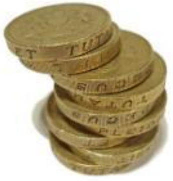

intense financial pressures

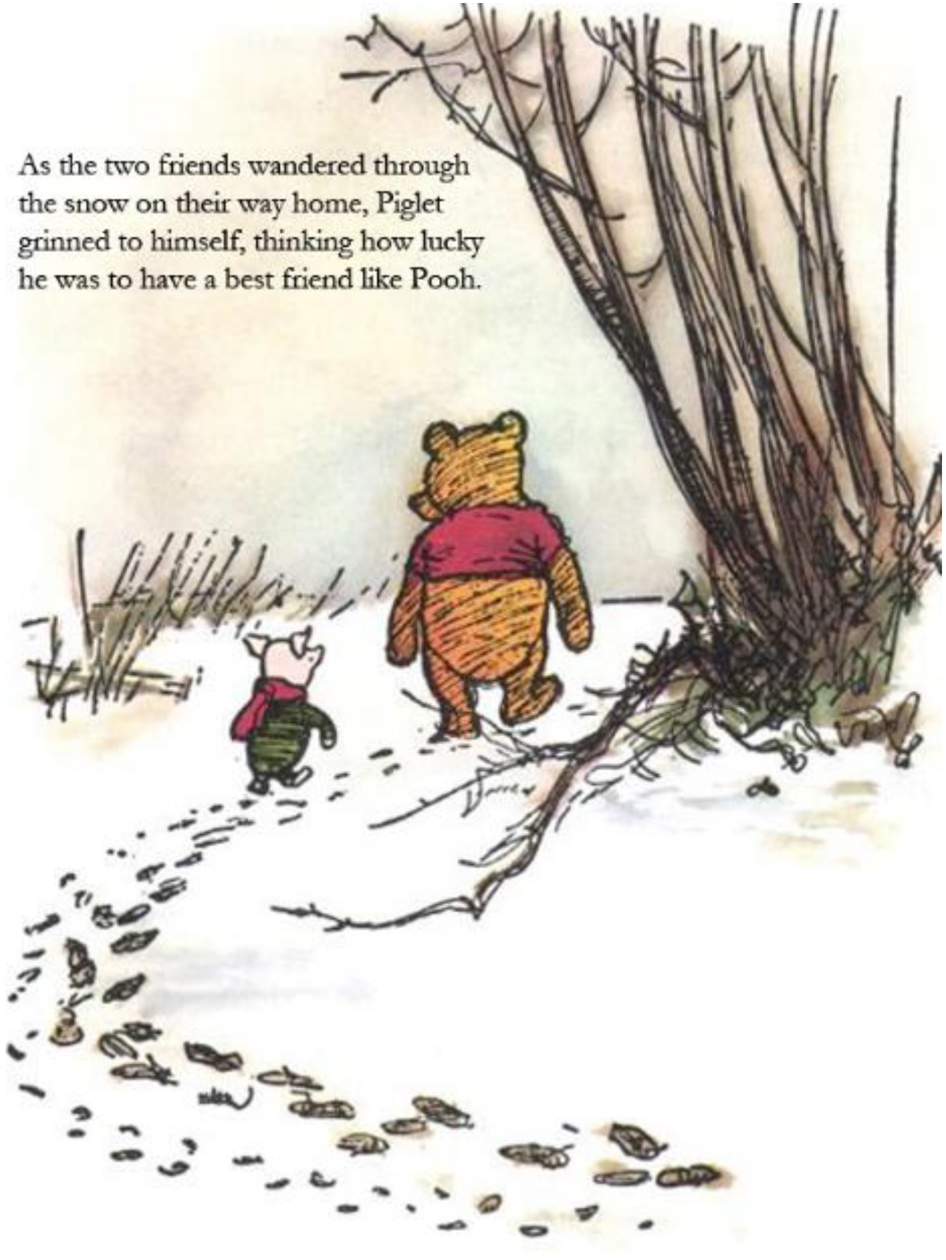
As the two friends wandered through the snow on their way home, Piglet grinned to himself, thinking how lucky he was to have a best friend like Pooh.

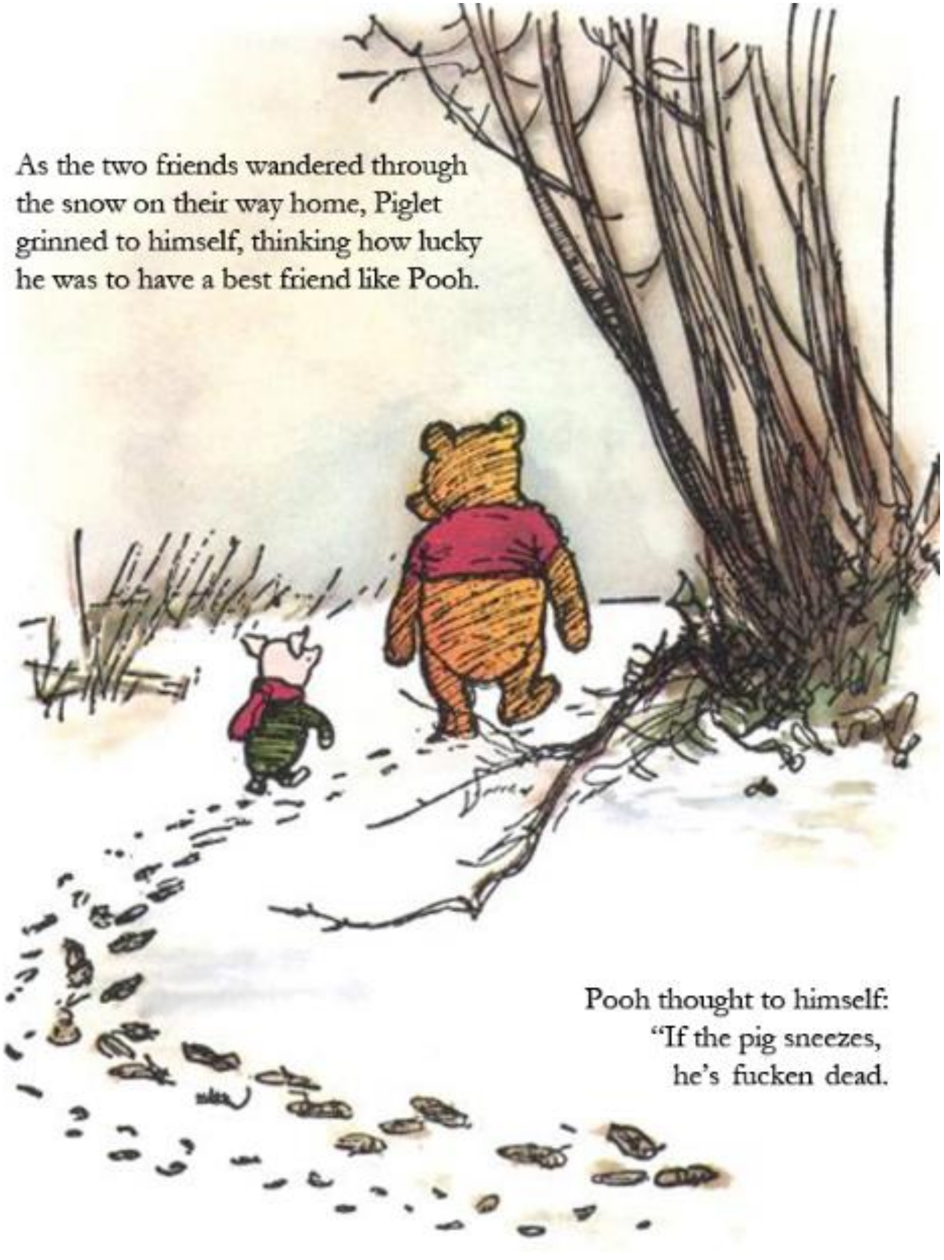
As the two friends wandered through the snow on their way home, Piglet grinned to himself, thinking how lucky he was to have a best friend like Pooh.
Pooh thought to himself: “If the pig sneezes, he’s fucken dead.

Academic Health Sciences Centres

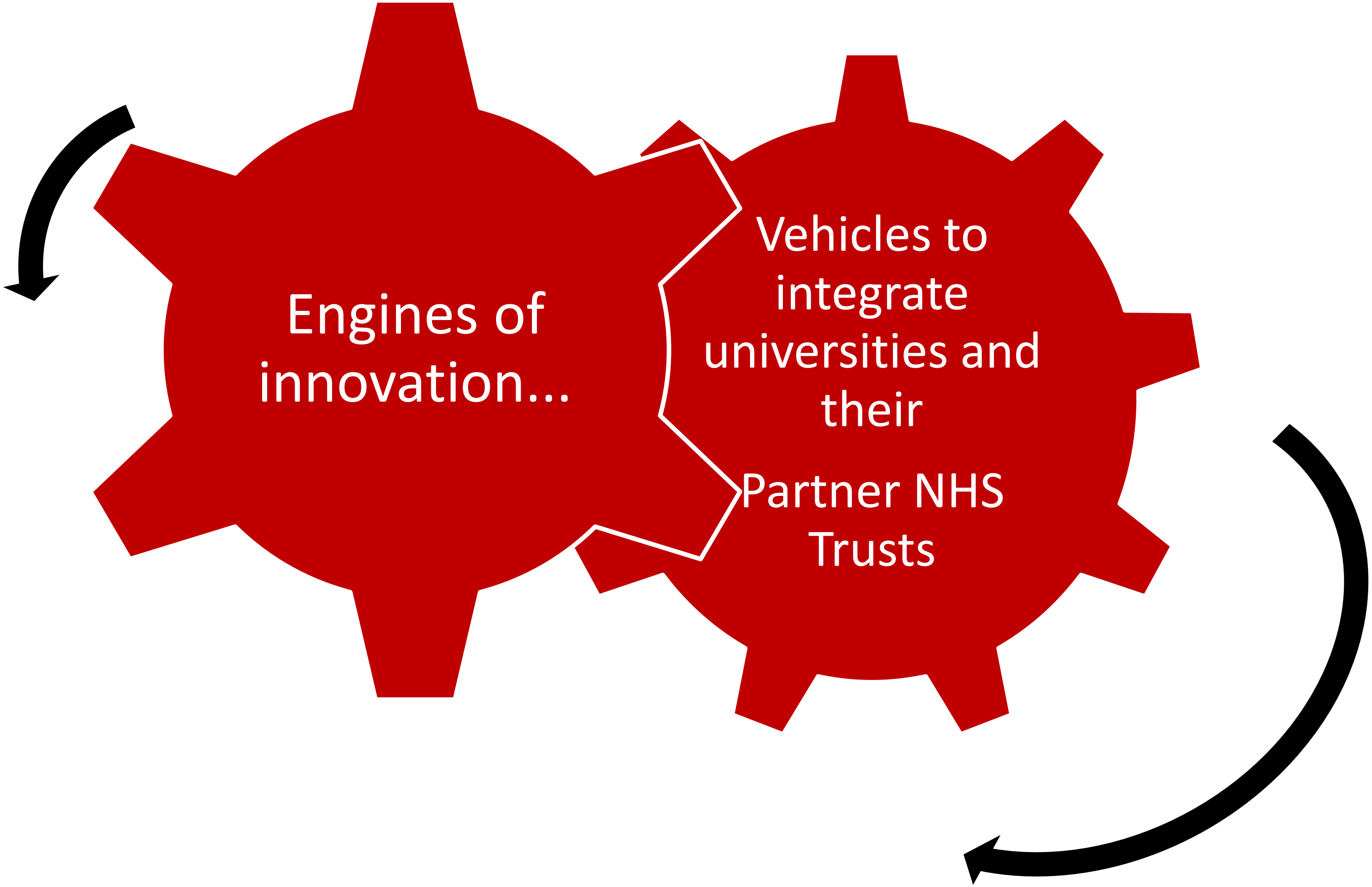

King's Health Partners: Excellence, breadth and scale

Four highly-performing institutions

- Guy's and St Thomas' (GSTT)
- King's College Hospital (KCH)
- South London and Maudsley (SLaM)
- King's College London (KCL)

Excellence in clinical service

- Comprehensive portfolio of excellent quality innovative services
- International recognition: in renal and liver disease, dermatology, haematology, children's, neurosciences, fetal medicine and mental health

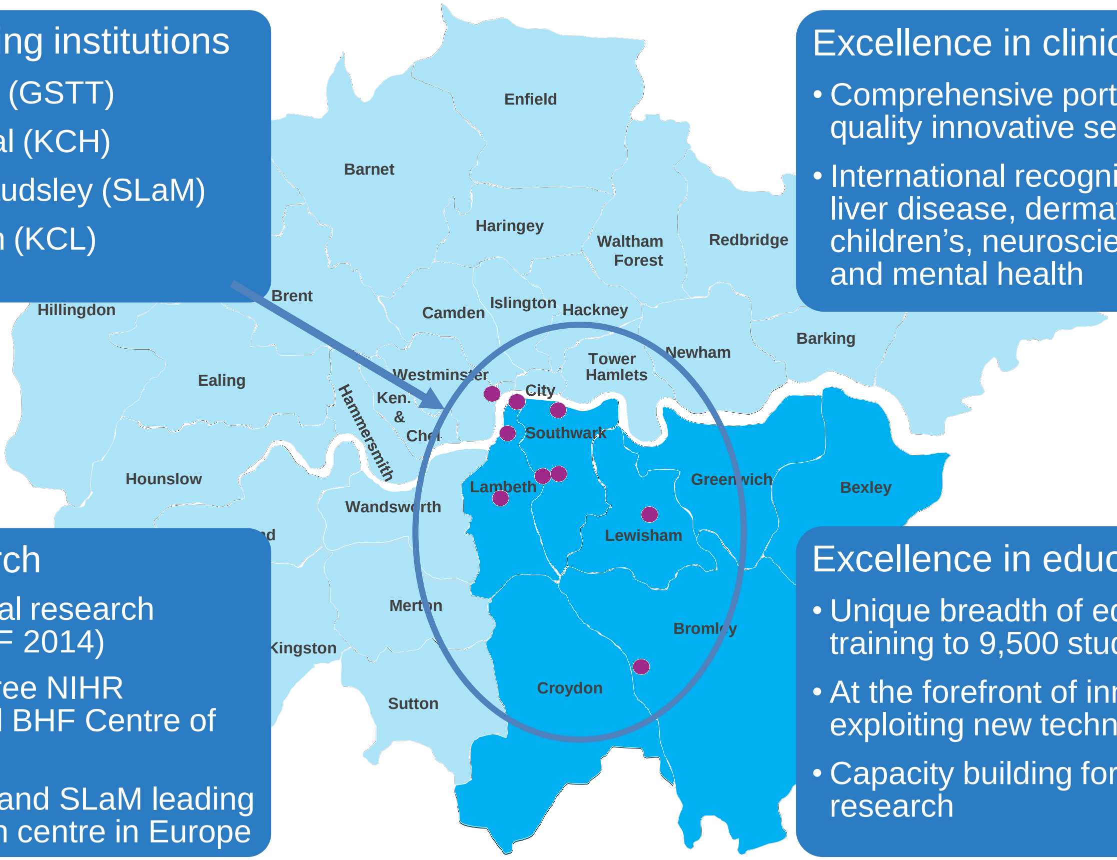

Excellence in research

- One of top 5 biomedical research universities in UK (REF 2014)
- Five MRC Centres, three NIHR Research Centres and BHF Centre of Excellence
- Institute of Psychiatry and SLaM leading mental health research centre in Europe

Excellence in education

- Unique breadth of education and training to 9,500 students
- At the forefront of innovation and exploiting new technology
- Capacity building for translational research

36,000 staff; turnover of £3.7bn p.a.

The three hypotheses underlying King's Health Partners

Two acute Trusts… and a mental health Trust and a university

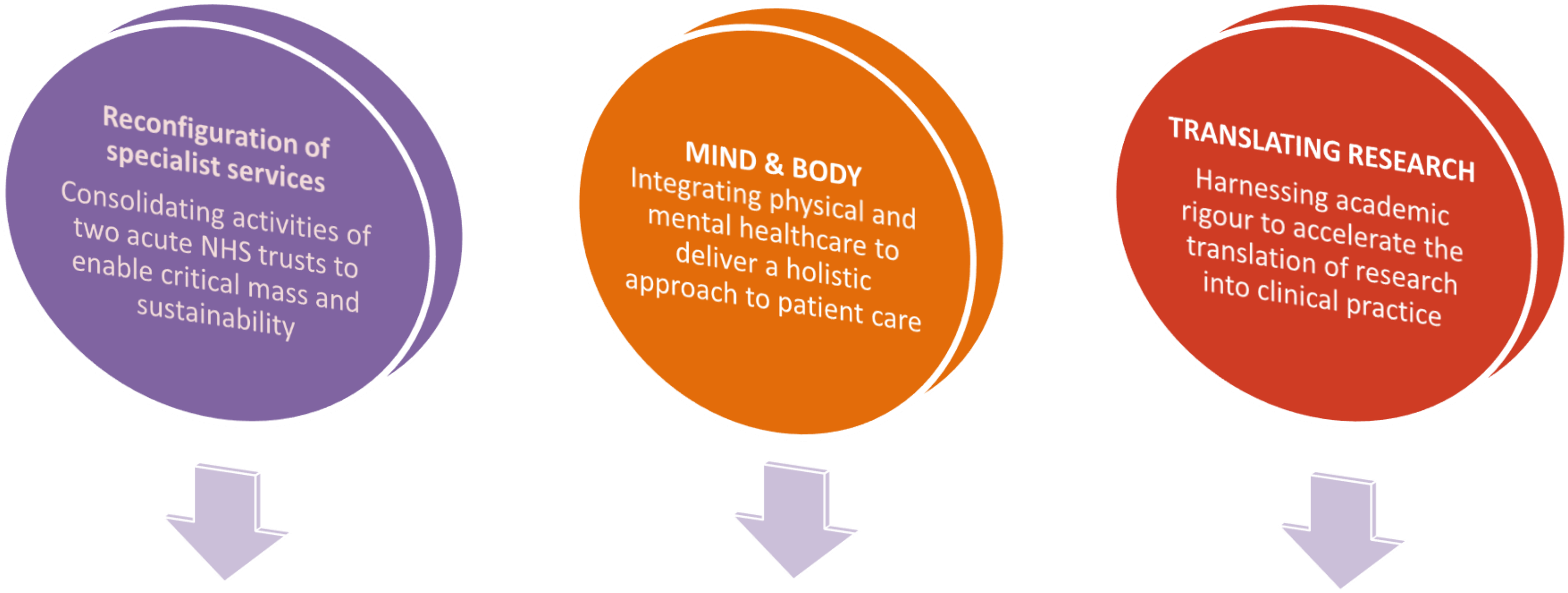

Increase in quality and impact on population health

The picture today (dog's breakfast)

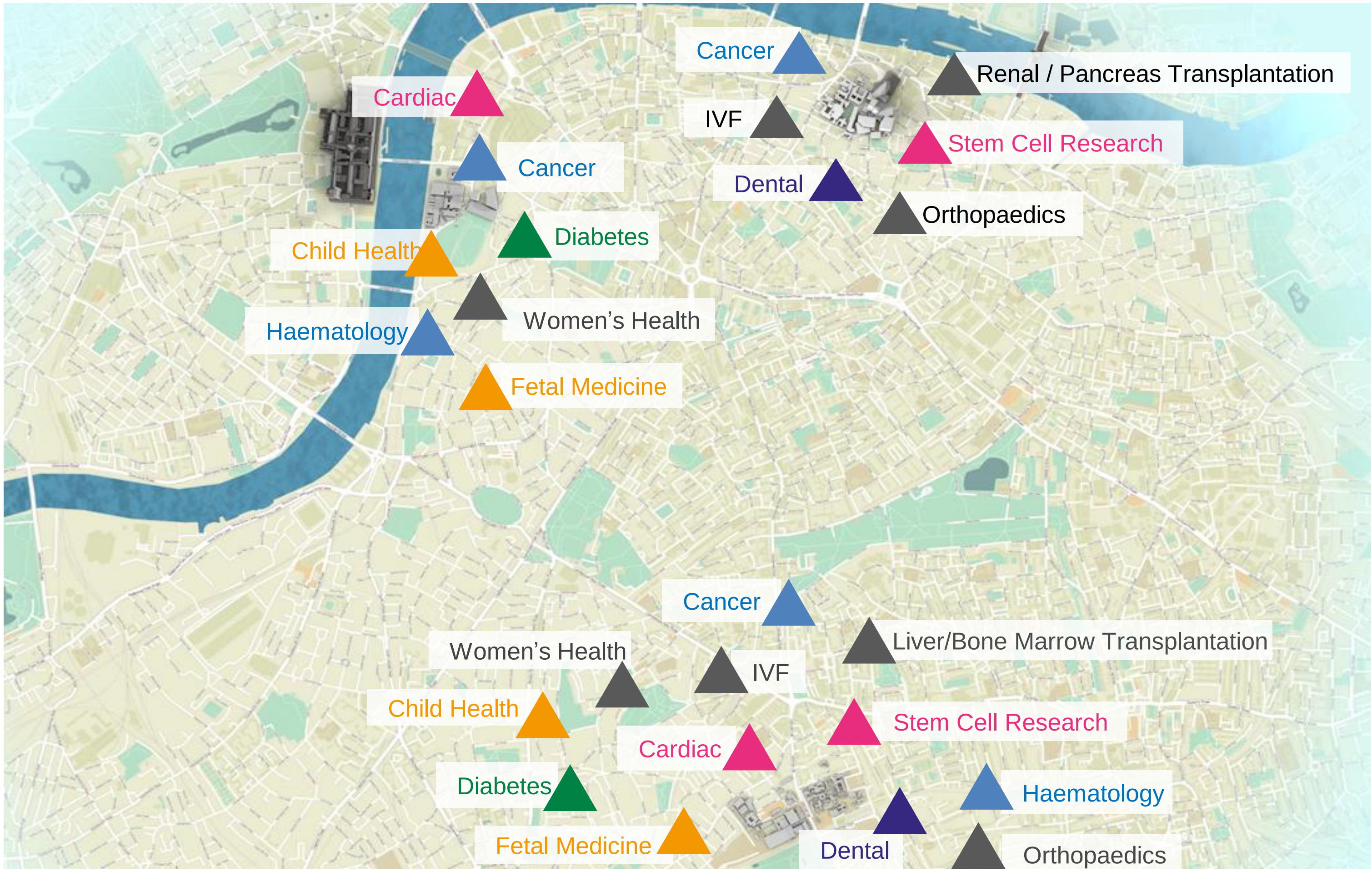

Clinical:academic institutes to deliver excellence

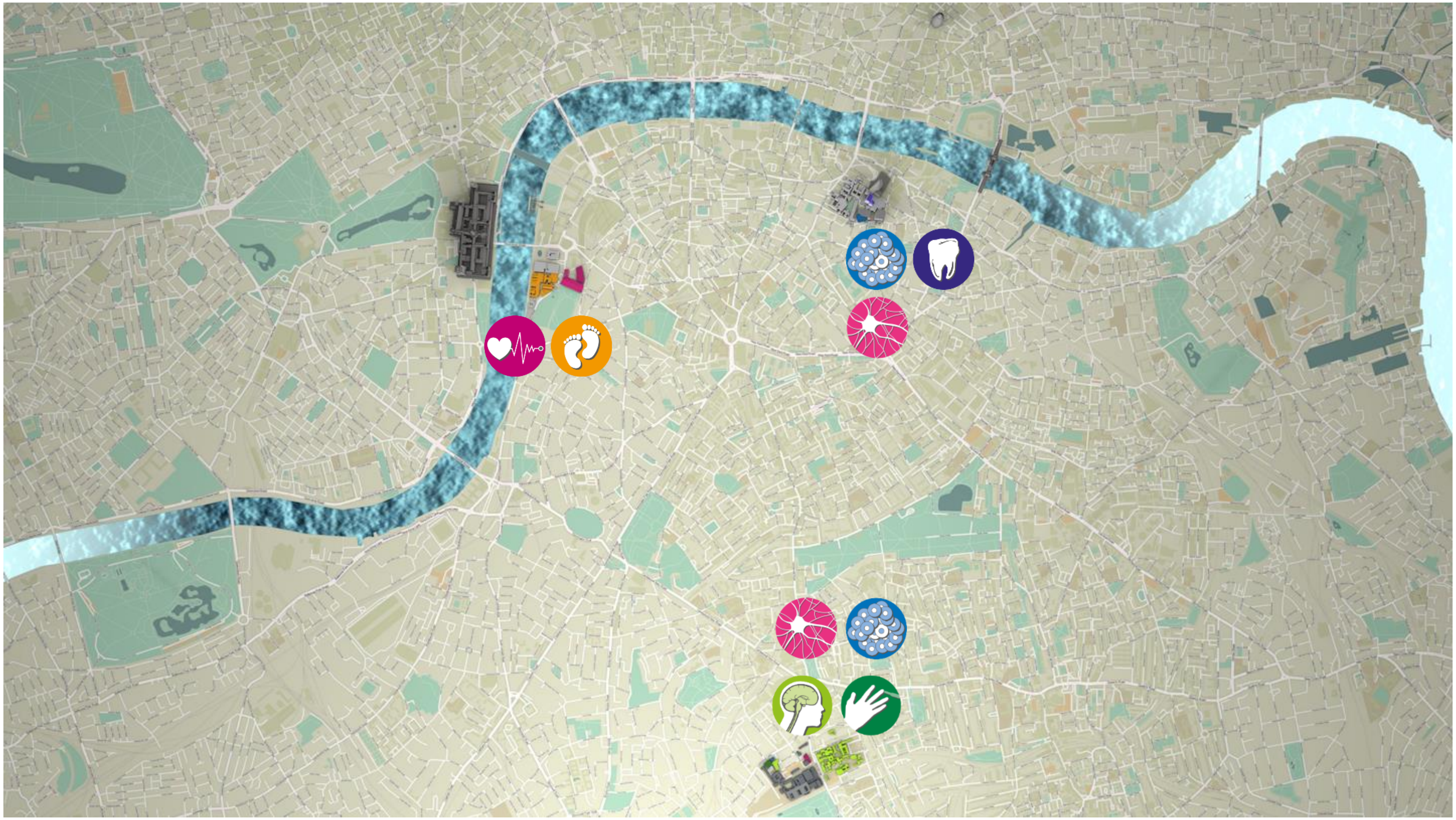

The Geography of Cartesian Dualism…

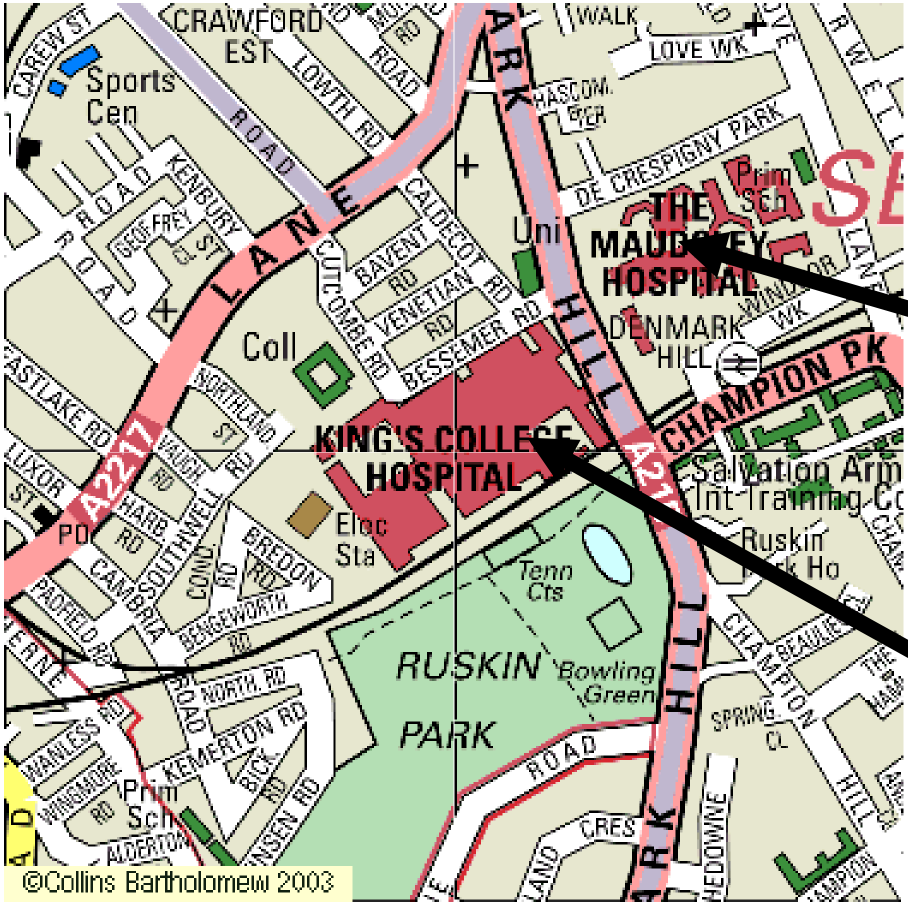

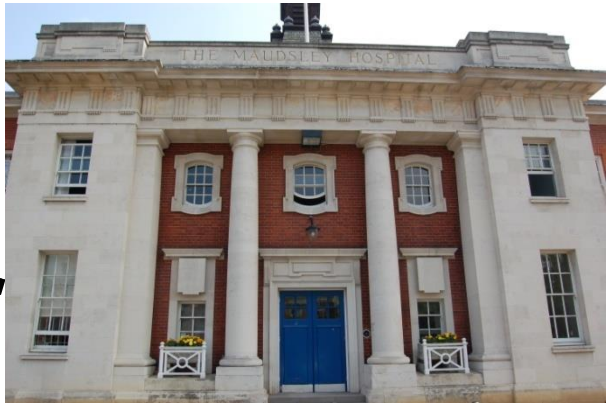

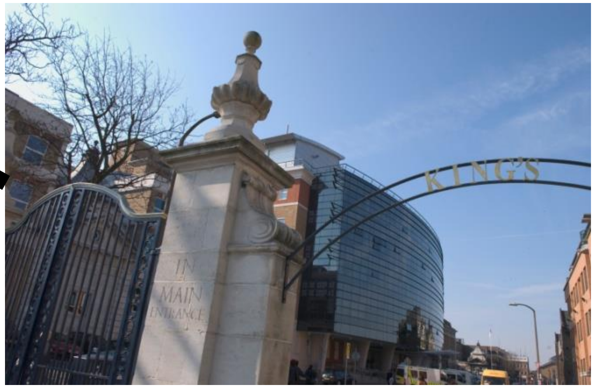

The challenge

- 30% patients with long term conditions are depressed
- 60% patients referred to a cardiologist with chest pain have nothing wrong with their heart
- Patients with long term serious mental illness die 17 years prematurely

Integrating Mental & Physical healthcare: Research, Training & Services

imparts
Integrating Mental & Physical healthcare: Research, Training & Services

Depression is a common condition for patients with physical illnesses – for example, affecting 23% of rheumatology patients.

Anxiety is similarly common, for example affecting 21% of orthopaedics patients.

- ➢ 74,433 screenings completed.
- ➢ Available in 58 outpatient clinics across King's Health Partners.

IMPARTS clinics across King's Health Partners:

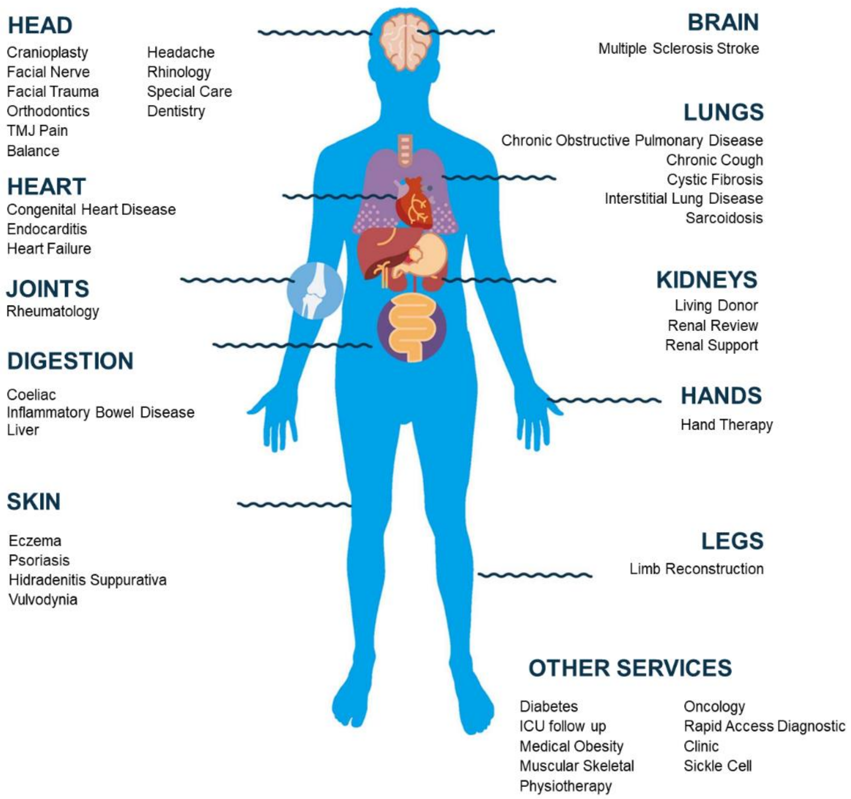

kingshealthpartners.org/value

Benefits of university-NHS partnerships

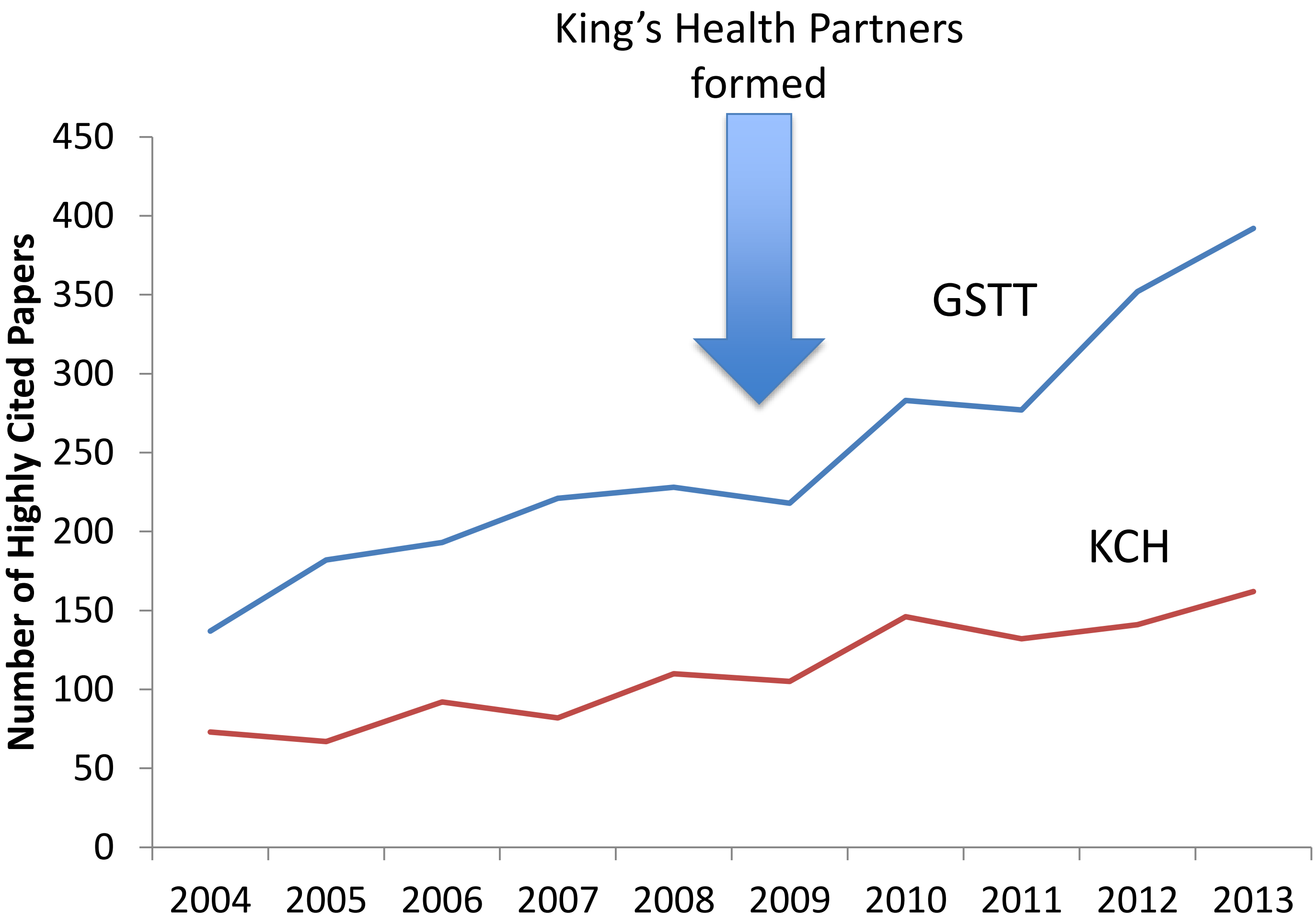

Clinical trials performance

All three of our trusts continually increase their year-on-year levels of clinical research studies, and are among the highest recruiting organisations in the country.

NIHR Research Activity League Table: acute trusts

Trust name	Type	Local network	Number of studies recruiting 2014/15	Number of studies recruiting 2015/16	Percentage change	Participants in studies 2014/15	Participants in studies 2015/16	Percentage change
GUY'S AND ST THOMAS' NHS FOUNDATION TRUST	Acute	South London	459	494 2nd	7.6%	23187	27813 1st	20.0%
OXFORD UNIVERSITY HOSPITALS NHS FOUNDATION TRUST	Acute	Thames Valley and South Midlands	411	463	12.7%	17827	21169	18.7%
UNIVERSITY HOSPITAL SOUTHAMPTON NHS FOUNDATION TRUST	Acute	Wessex	385	391	1.6%	20759	20939	0.9%
KING'S COLLEGE HOSPITAL NHS FOUNDATION TRUST	Acute	South London	311	337 11th	8.4%	12520	14776 4th	18.0%

NIHR Research Activity League Table: mental health trusts

Trust name	Type	Local network	Number of studies recruiting 2014/15	Number of studies recruiting 2015/16	Percentage change	Participants in studies 2014/15	Participants in studies 2015/16	Percentage change
SOUTH LONDON AND MAUDSLEY NHS FOUNDATION TRUST	Mental Health	South London	91	94 1st	3.3%	2717	2527 3rd	-7.0%
MANCHESTER MENTAL HEALTH AND SOCIAL CARE TRUST	Mental Health	Greater Manchester	42	52	23.8%	933	1187	27.2%
OXFORD HEALTH NHS FOUNDATION TRUST	Mental Health	Thames Valley and South Midlands	40	48	20.0%	2102	2576	22.5%

Keys to accelerating translation

- Maintaining a balanced science base
- Fostering university – healthcare partnerships
- Establishing a sustainable healthcare system
- Fostering partnerships with industry
- Attracting the most able scientists, clinical and non-clinical, into biomedical research careers
- Building the infrastructure for “experimental medicine”
- Anticipating the evolution of healthcare – the digital revolution
- Effective public engagement

Putting Value-based Healthcare into practice

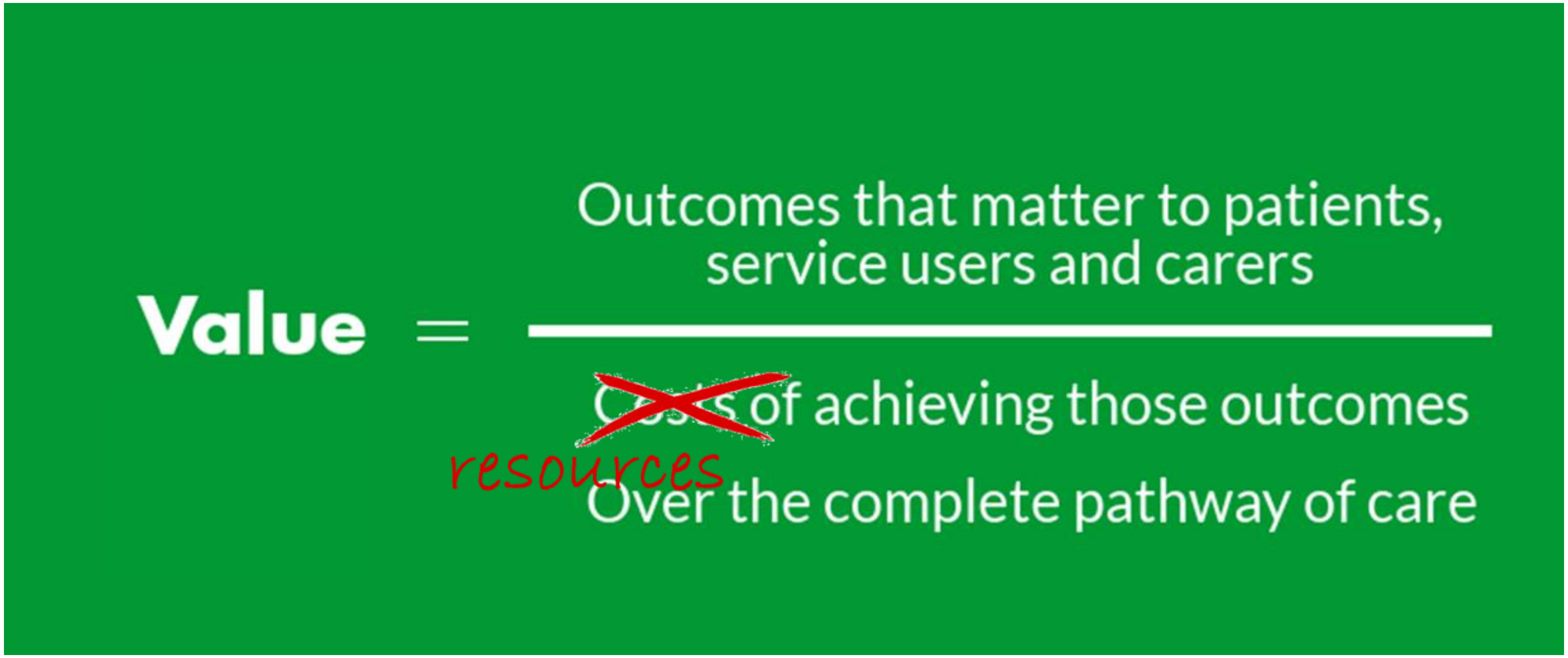

CEBM

Value-based healthcare is the equitable, sustainable and transparent use of the available resources to achieve better outcomes and experiences for every person.

“Defining Value-based Healthcare in the NHS”, Hurst L, Mahtani K, Pluddemann A, Lewis S, Harvey K, Briggs A, Boylan A-M, Bajwa R, Haire K, Entwistle A, Handa A, and Heneghan C. CEBM, University of Oxford (April 2019). https://www.cebm.net/2019/04/defining-value-based-healthcare-in-the-nhs/

KHP has a broad programme of work in Value Based Healthcare

A value-driven NHS can only be achieved through sharing and use of outcomes that matter to patients and clinically-meaningful cost information

Outcomes Books

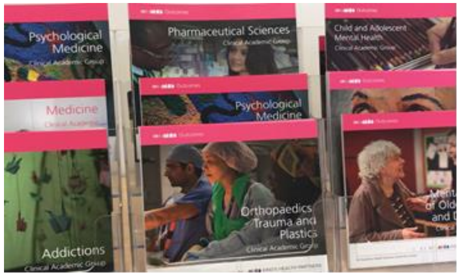

Outcomes Scorecards

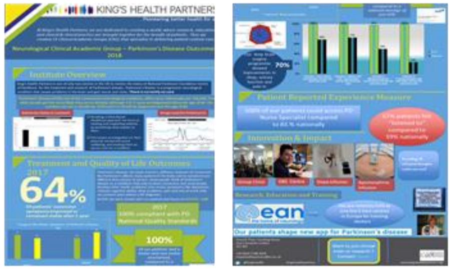

Calculating Value

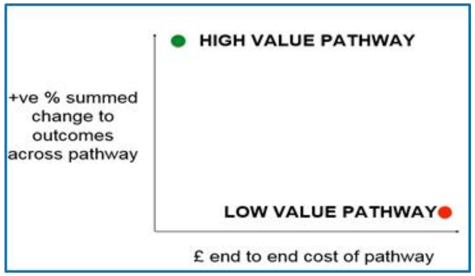

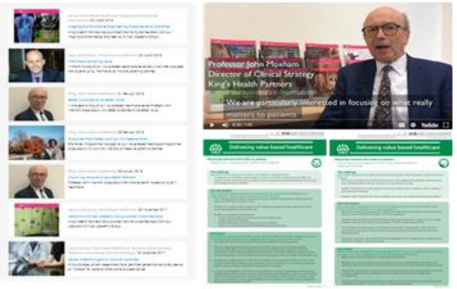

Communications

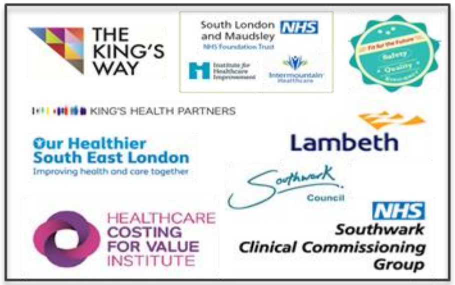

Sharing learning

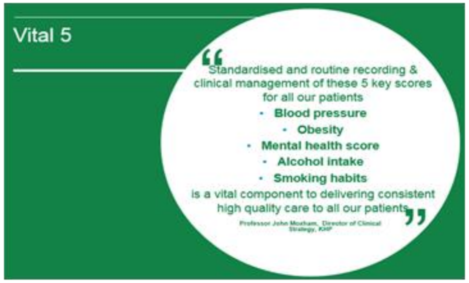

Vital 5

Categories of value-creating interventions

1. Service reconfiguration
2. Pathway redesign
3. Frugal innovation
4. Prevention

Keys to accelerating translation

- Maintaining a balanced science base
- Fostering university – healthcare partnerships
- Establishing a sustainable healthcare system
- Fostering partnerships with industry
- Attracting the most able scientists, clinical and non-clinical, into biomedical research careers
- Building the infrastructure for “experimental medicine”
- Anticipating the evolution of healthcare – the digital revolution
- Effective public engagement

A broken model of drug development

Large pharma annual spend c.$156bn and increasing
www.abpi.org.uk/facts-and-figures/science-and-innovation/worldwide-pharmaceutical-company-rd-expenditure/

Only c.26 new molecular entities (drugs) approved by the FDA in 2018 – but 59 including mAbs and advanced therapies

Attrition from phase 1 to market launch ~90%

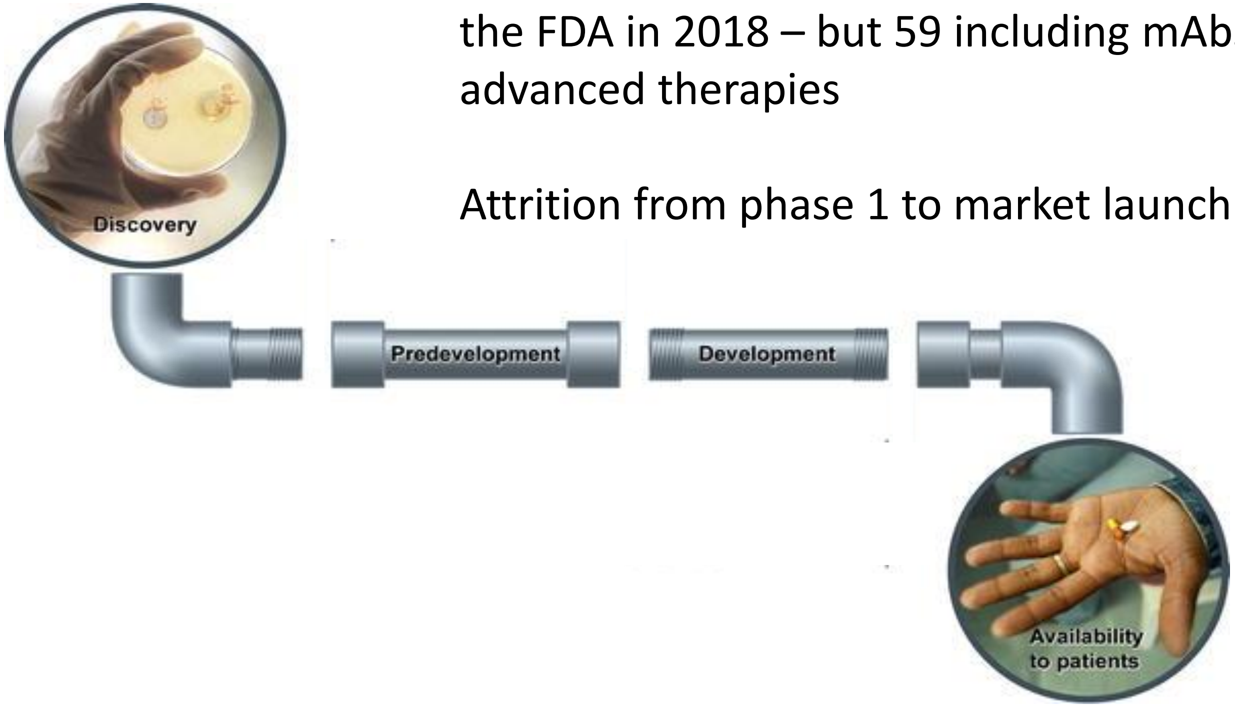

R&D Productivity Gap

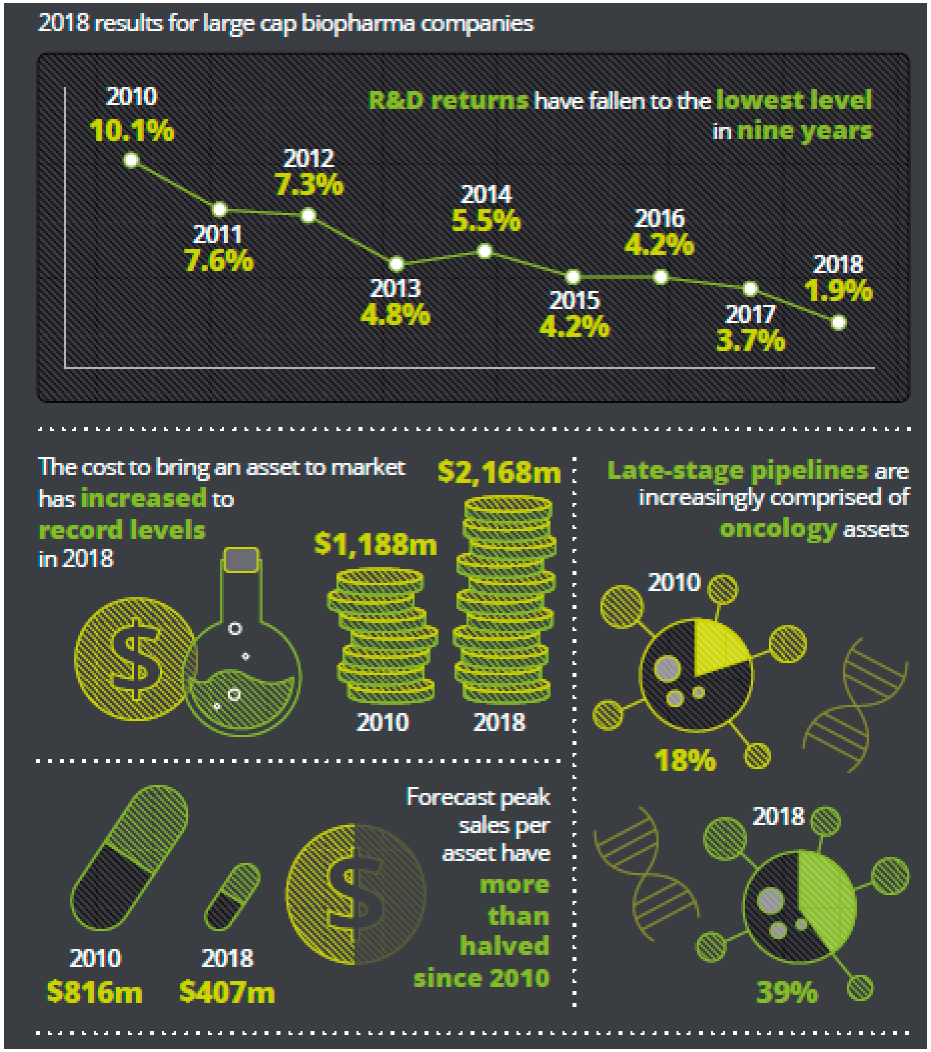

www2.deloitte.com/uk/en/pages/life-sciences-and-healthcare/articles/measuring-return-from-pharmaceutical-innovation.html

New partnership models

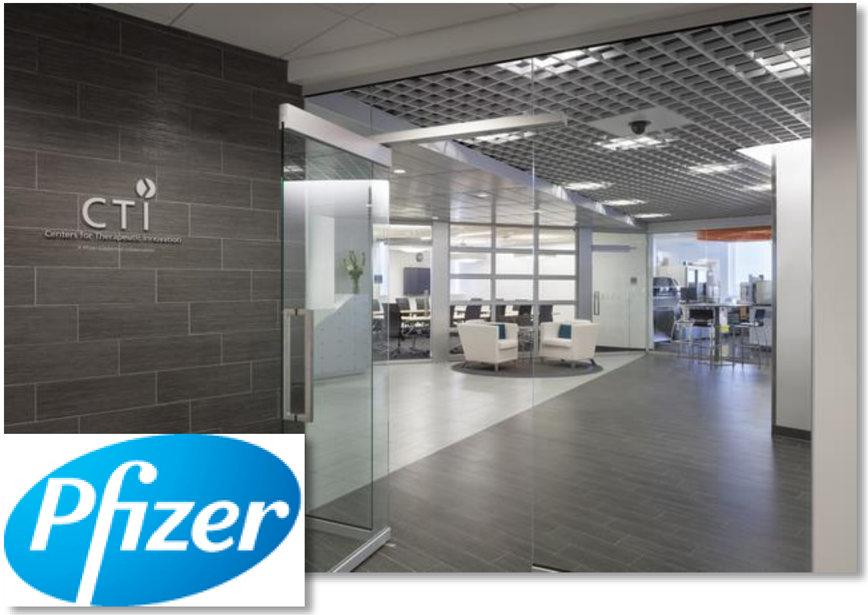

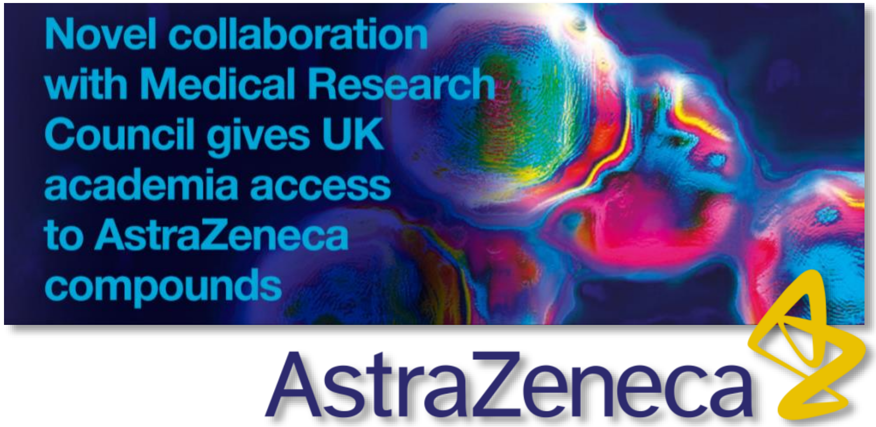

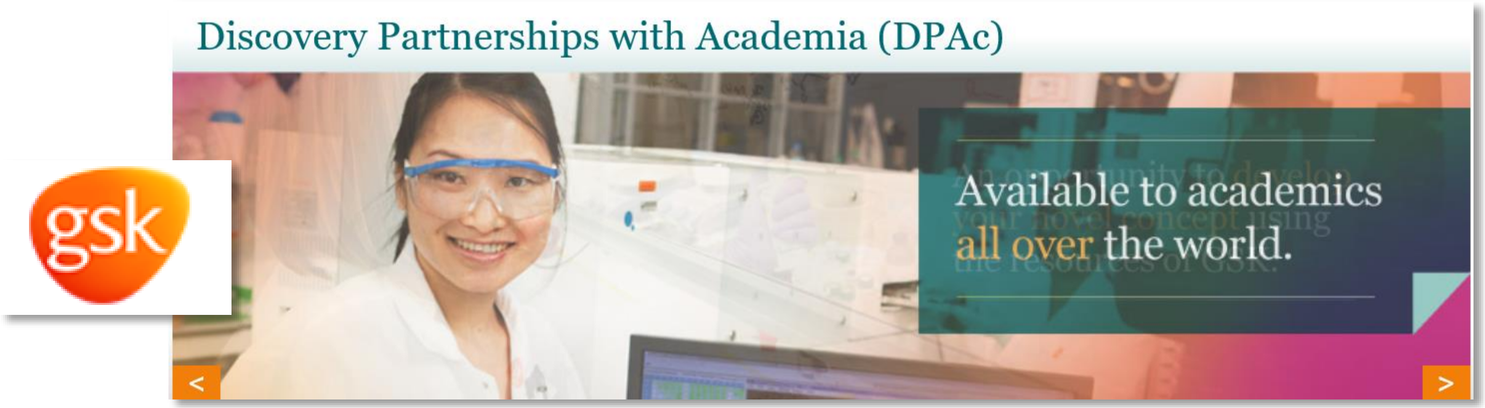

University-NHS-Industry interactions

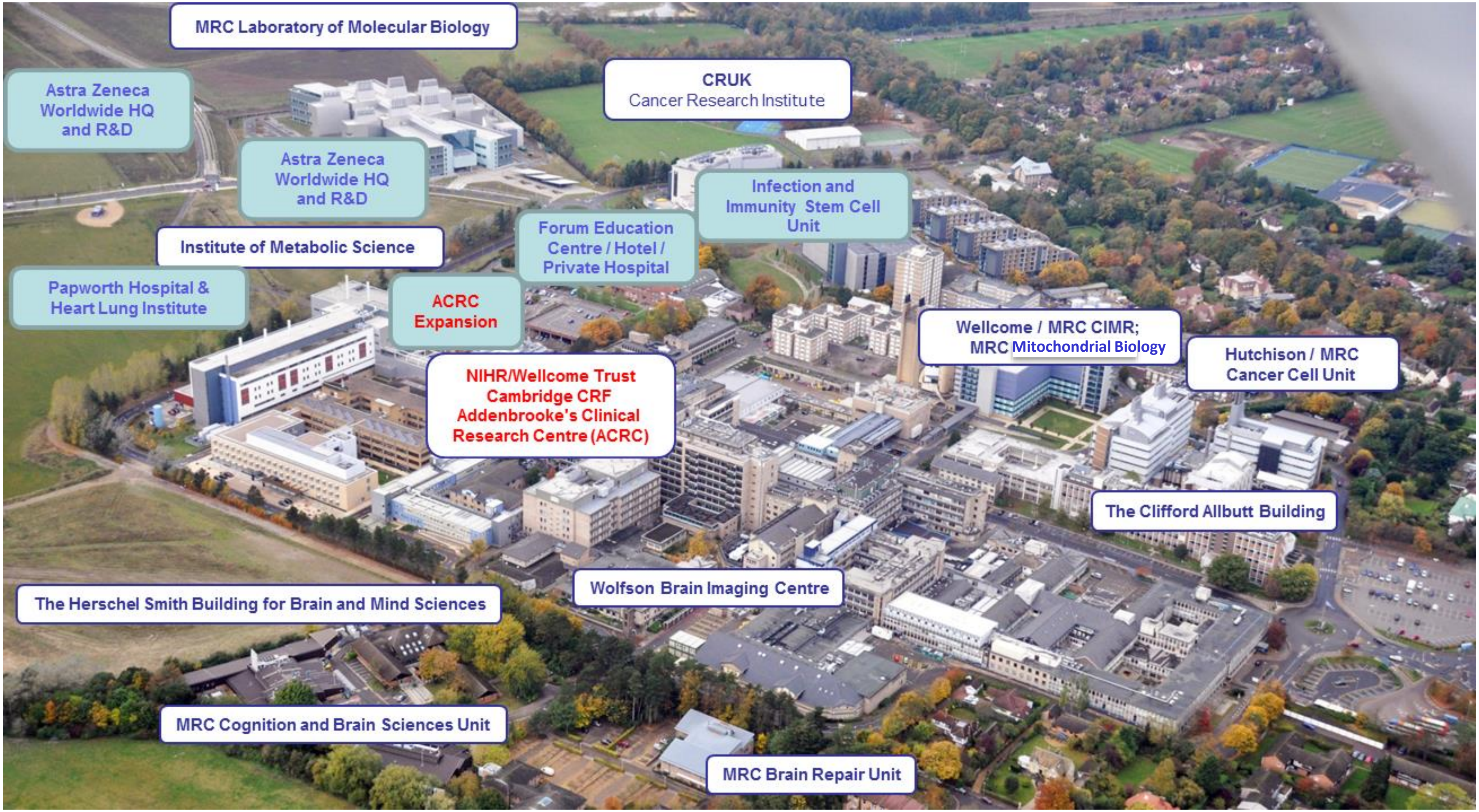

Ways to foster closer collaboration

- More porous boundary between academe and industry
- *UG curriculum*
- *Internships*
- *Sabbaticals*
- *Movement as part of an accepted career path (not one-way street)*

Success in translation of discovery and innovation into patient benefit depends on partnerships

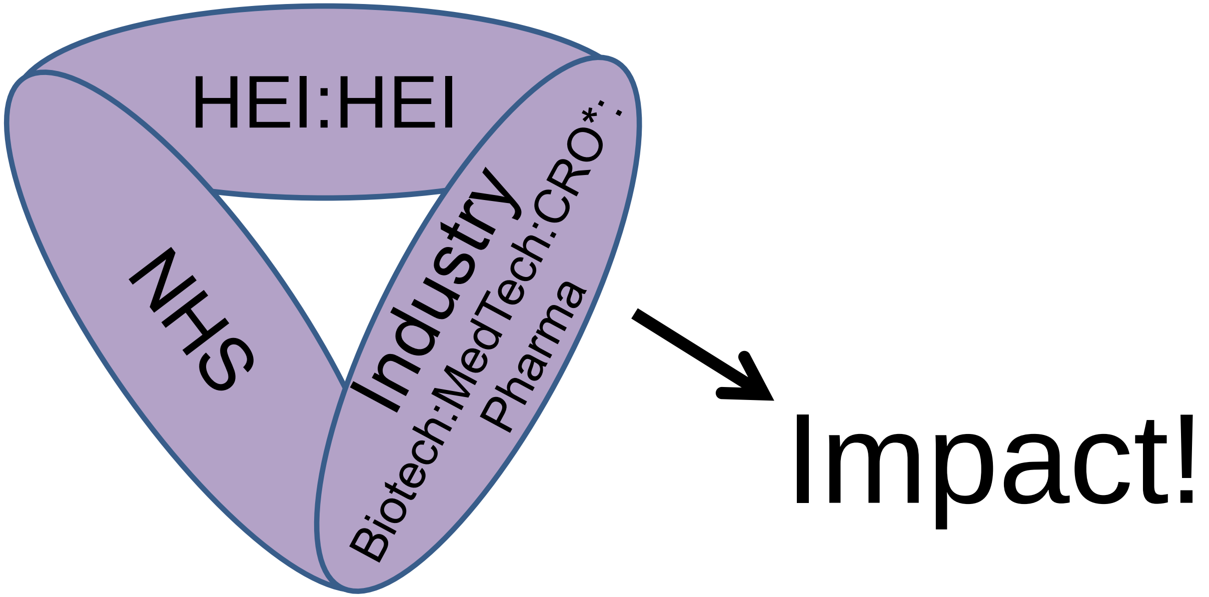

A tri-partite relationship to deliver a tri-partite mission

*CRO, contract research organization

Collaborative clusters to ensure international competitiveness

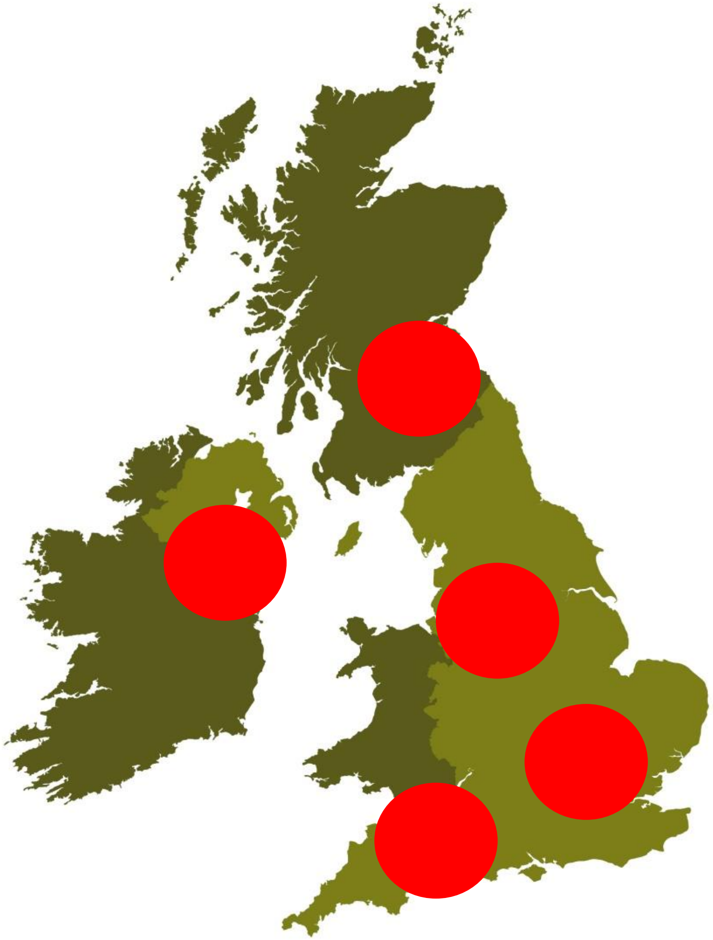

Keys to accelerating translation

- Maintaining a balanced science base
- Fostering university – healthcare partnerships
- Establishing a sustainable healthcare system
- Fostering partnerships with industry
- Attracting the most able scientists, clinical and non-clinical, into biomedical research careers
- Building the infrastructure for "experimental medicine"
- Anticipating the evolution of healthcare – the digital revolution
- Effective public engagement

Gender and Equality

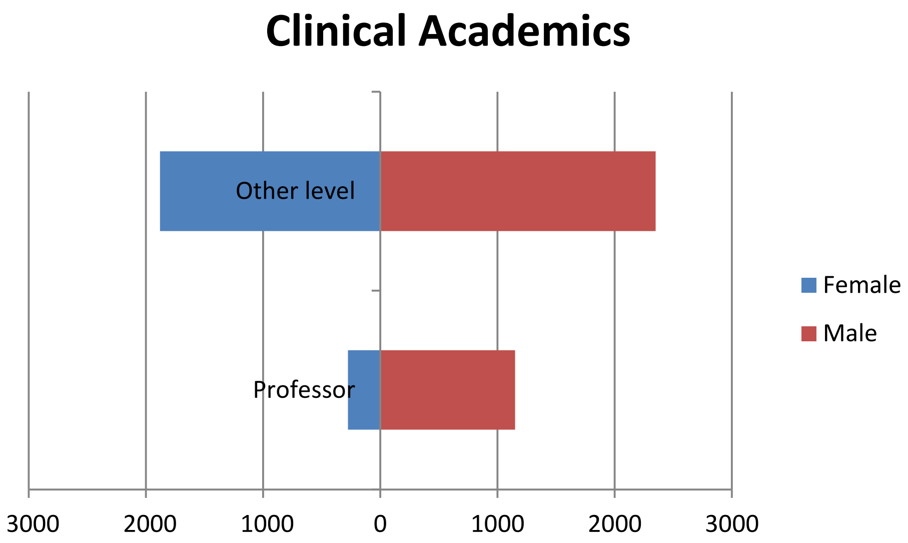

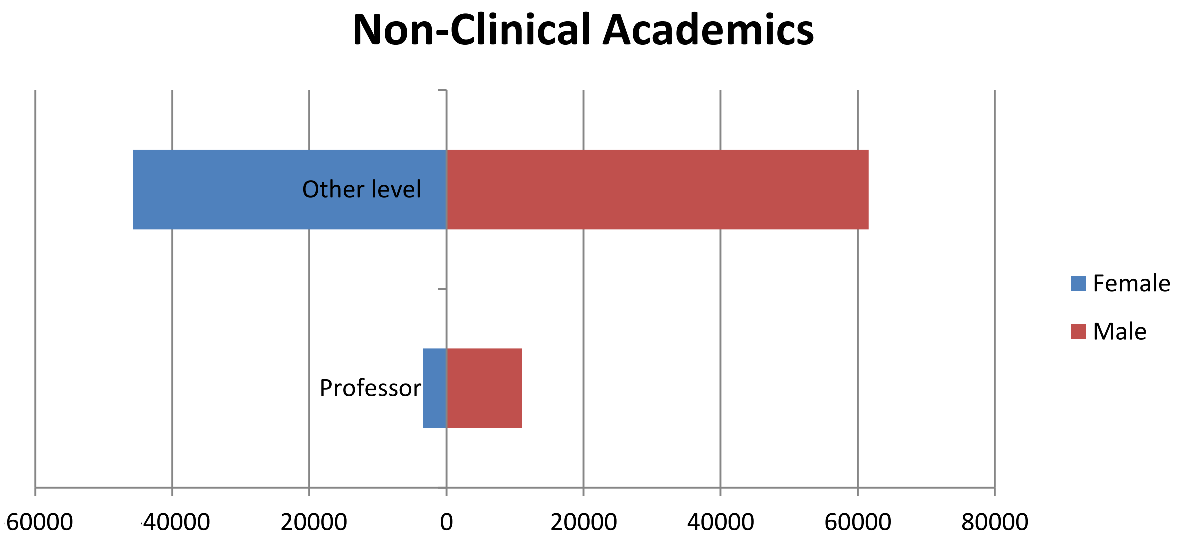

Equality and Diversity

Shortage of women in the professoriate

The best minds from all backgrounds

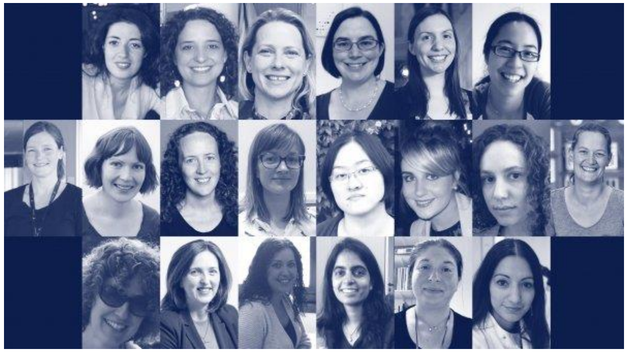

Mentorship – AMS SUSTAIN programme

Keys to accelerating translation

- Maintaining a balanced science base
- Fostering university – healthcare partnerships
- Establishing a sustainable healthcare system
- Fostering partnerships with industry
- Attracting the most able scientists, clinical and non-clinical, into biomedical research careers
- Building the infrastructure for “experimental medicine”
- Anticipating the evolution of healthcare – the digital revolution
- Effective public engagement

Experimental Medicine Hub in Guy's Tower

Facility	Floor
Centre for Stem Cells and Regenerative Medicine	28
Dental Institute Clinical Research Unit	25
Dental Institute Centre for Innovation and Translation	17
BRC, R&D, Data Analytics	16
NIHR Clinical Research Facility, BRC Immune Monitoring Platform, BRC Advanced Therapy Manufacturing (GMP) Facility	15
BRC Commercialisation and Innovation Hub*	14
GMP Pharmacy Manufacturing Unit	13
Cell and Gene Therapy Catapult	12
Assisted Conception Unit and Embryonic Stem Cell Facility	11
BRC Advanced Therapeutics Centre*	10
BRC Genomics Platform	7
Retroviral Manufacturing Unit	4
Intensive Care Unit	1

*Proposed/planned new facilities

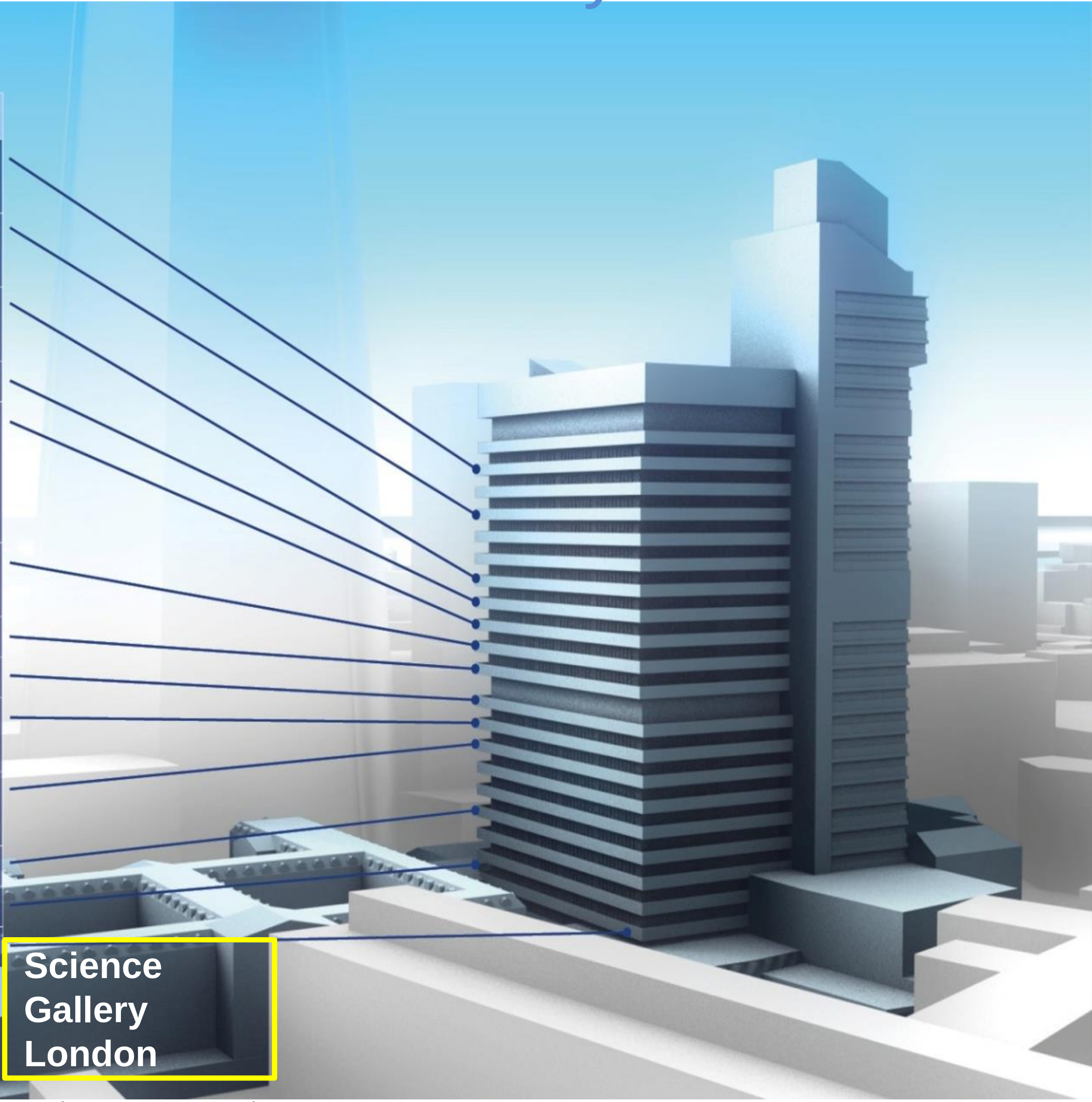

Experimental Medicine Infrastructure at St Thomas'

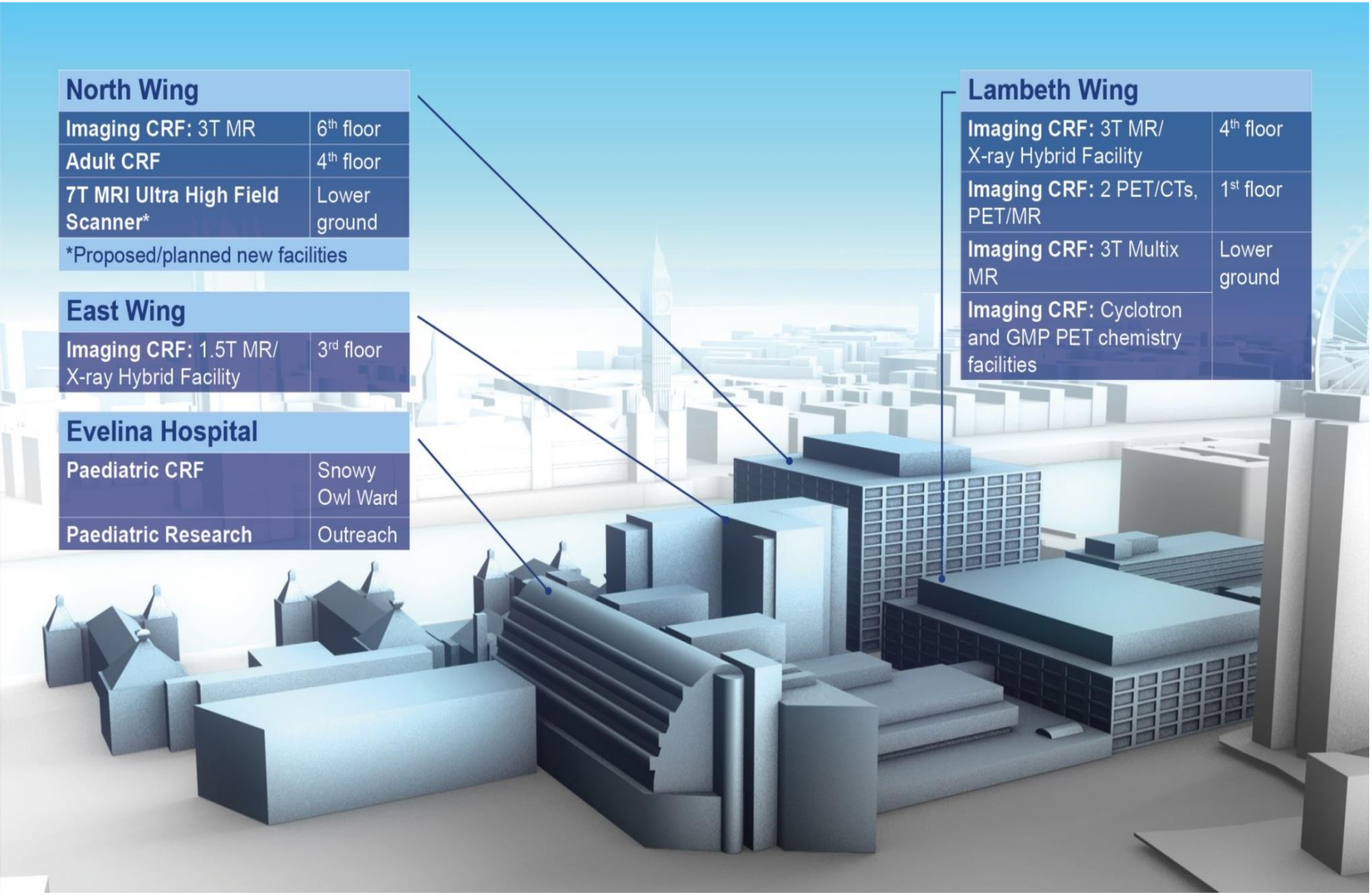

www.guysandstthomasbrc.nihr.ac.uk

Keys to accelerating translation

- Maintaining a balanced science base
- Fostering university – healthcare partnerships
- Establishing a sustainable healthcare system
- Fostering partnerships with industry
- Attracting the most able scientists, clinical and non-clinical, into biomedical research careers
- Building the infrastructure for “experimental medicine”
- Anticipating the evolution of healthcare – the digital revolution
- Effective public engagement

Digital Revolution in Health and Social Care

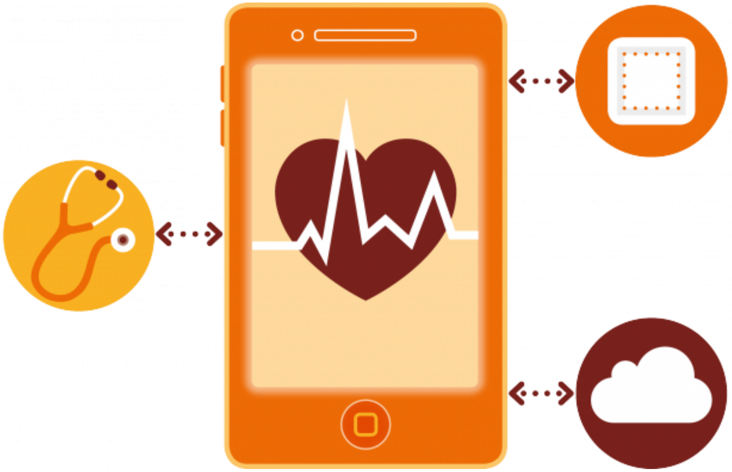

- Smartphones/apps/monitoring
- Smart pills/implanted devices
- Remote diagnostics (Peek Vision)
- Research data collection

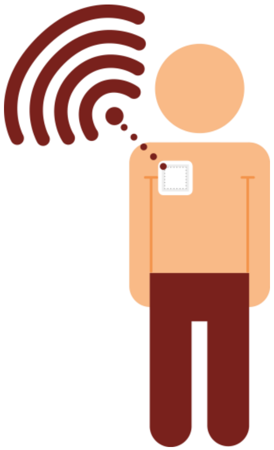

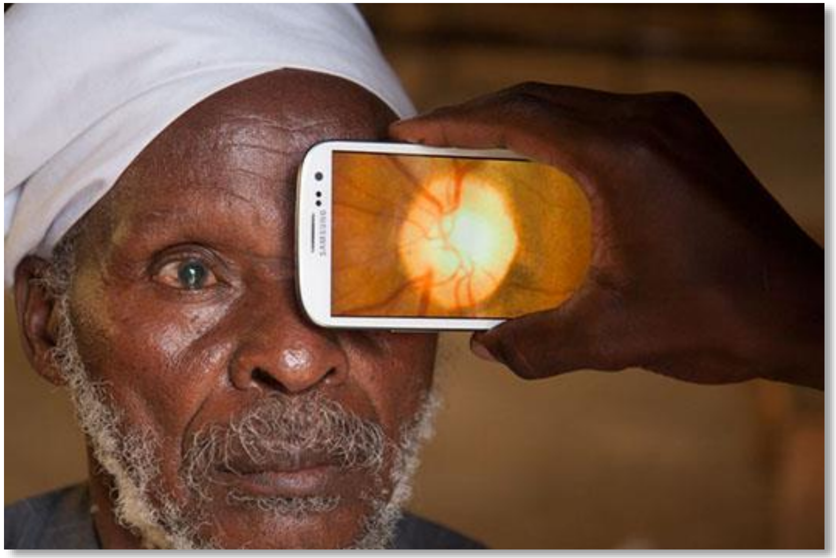

The King’s Fund>

Digital Revolution in Health and Social Care

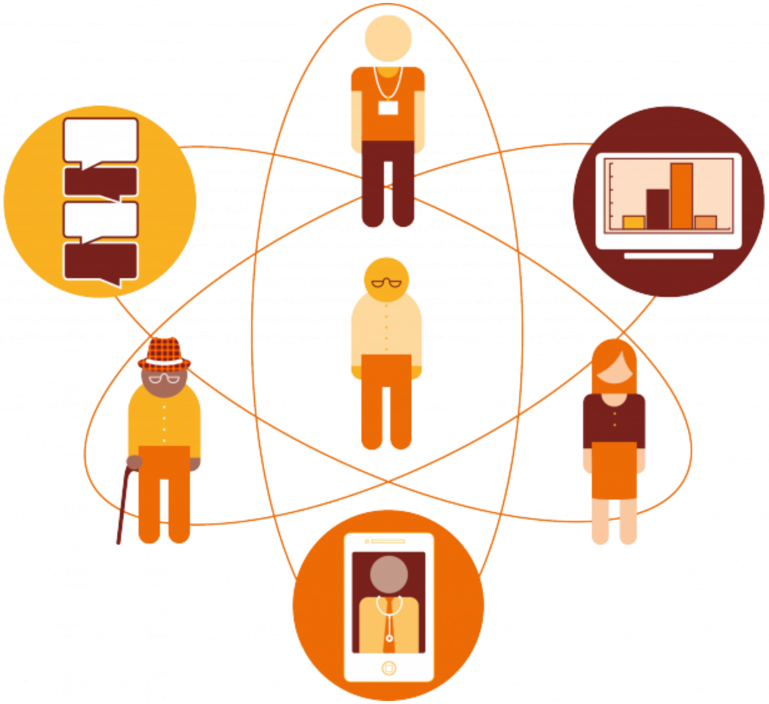

- Connectivity across services and individuals
- Personal ownership of health records
- Patient empowerment

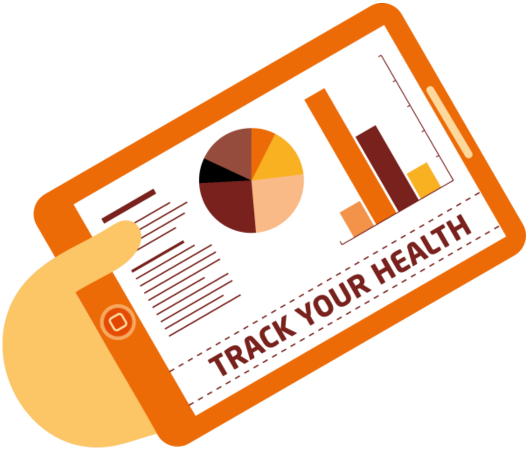

The King's Fund>

Digital Revolution in Health and Social Care

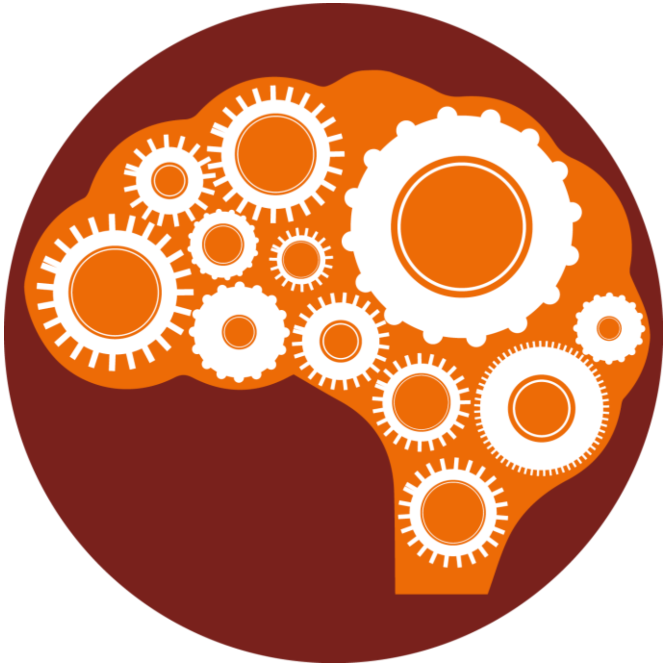

- Machine learning
- Artificial intelligence
- Big data
- Analysis of electronic health records

The King’s Fund>

Need to prepare for a radical change in healthcare delivery

Need for new research methodologies, particularly for bringing in non-biomedical sciences

Implications for workforce?

Keys to sustaining success

- Maintaining a balanced science base
- Fostering university – NHS partnerships
- Establishing a sustainable healthcare system
- Fostering partnerships with industry
- Attracting the most able scientists, clinical and non-clinical, into biomedical research careers
- Anticipating the evolution of healthcare – the digital revolution
- Effective public engagement

Public Engagement

- Engaging the next generation, untapped groups

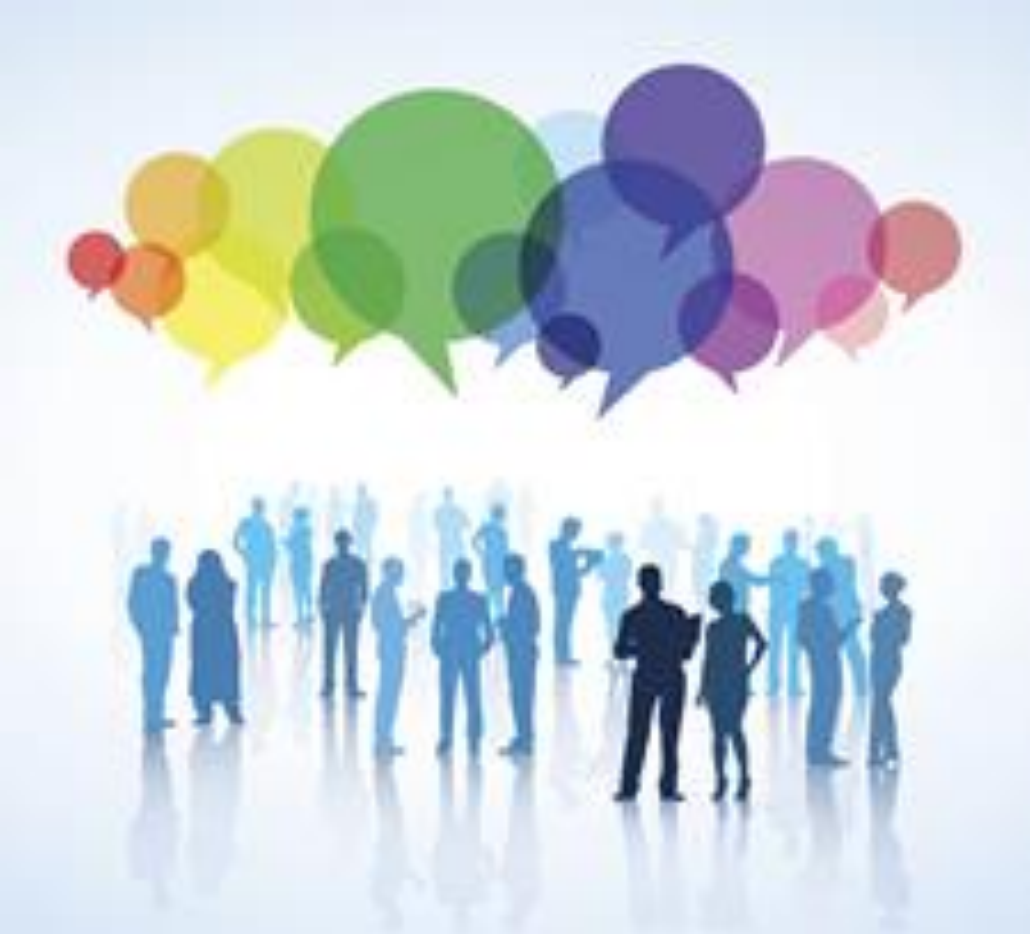

Need for "permission" from the public to pursue research goals – the tax payer is our funder

- Horizon scanning on future policy issues arising from science – e.g. CRISPR/Cas, stratified medicine

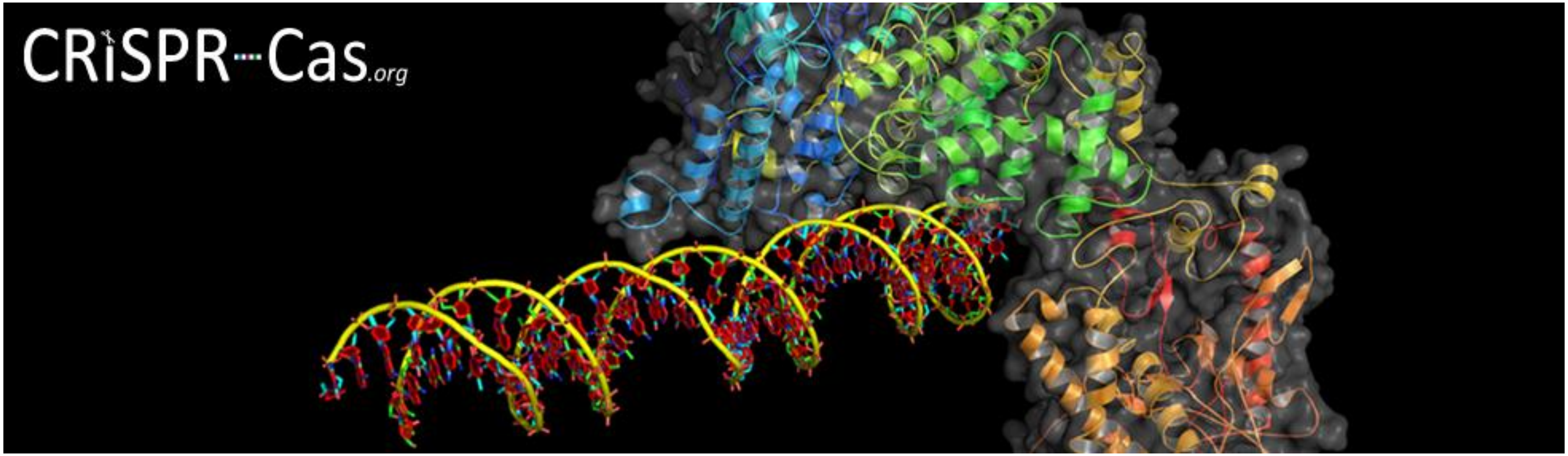

In conclusion:

- Resource discovery science
- Build on AHSC model to create line of sight culture
- Bring academic rigour to healthcare sustainability challenge
- Create more porous boundaries between academe and industry
- Support careers of young scientists
- Address the gender gap
- Invest in key infrastructure for experimental medicine and the digital revolution
- Prioritise public engagement
- Demonstrate economic value of research
